# Vision for the blind: visual psychophysics and blinded inference for decision models

**DOI:** 10.3758/s13423-020-01742-7

**Published:** 2020-06-08

**Authors:** Philip L. Smith, Simon D. Lilburn

**Affiliations:** Melbourne School of Psychological Sciences, The University of Melbourne, Victoria, 3010 Australia

**Keywords:** Evidence accumulation, Diffusion model, Selective influence, Random dot motion

## Abstract

Evidence accumulation models like the diffusion model are increasingly used by researchers to identify the contributions of sensory and decisional factors to the speed and accuracy of decision-making. Drift rates, decision criteria, and nondecision times estimated from such models provide meaningful estimates of the quality of evidence in the stimulus, the bias and caution in the decision process, and the duration of nondecision processes. Recently, Dutilh et al. (*Psychonomic Bulletin & Review 26*, 1051–1069, 2019) carried out a large-scale, blinded validation study of decision models using the random dot motion (RDM) task. They found that the parameters of the diffusion model were generally well recovered, but there was a pervasive failure of selective influence, such that manipulations of evidence quality, decision bias, and caution also affected estimated nondecision times. This failure casts doubt on the psychometric validity of such estimates. Here we argue that the RDM task has unusual perceptual characteristics that may be better described by a model in which drift and diffusion rates increase over time rather than turn on abruptly. We reanalyze the Dutilh et al. data using models with abrupt and continuous-onset drift and diffusion rates and find that the continuous-onset model provides a better overall fit and more meaningful parameter estimates, which accord with the known psychophysical properties of the RDM task. We argue that further selective influence studies that fail to take into account the visual properties of the evidence entering the decision process are likely to be unproductive.

The ability to make fast and accurate decisions about stimuli in the environment is the hallmark of all cognitive systems. In humans and nonhuman animals alike, evidence accumulation models like the diffusion model (Ratcliff, 1978; Ratcliff & McKoon, 2008) have provided insights into the processes that determine the speed and accuracy of decision-making (Smith & Ratcliff, 2004). The attraction of such models, for both basic and applied researchers, is that their parameters have meaningful psychological interpretations. When estimated from data, the model parameters can help researchers understand which processes are affected by experimental manipulations and, in individual differences settings, the parameters can be interpreted psychometrically to help understand why one participant population differs from another (Ratcliff et al., 2015). The availability of third-party software packages for fitting the diffusion model to data, such as fast-dm (Voss & Voss, 2007), HDDM (Wiecki et al., 2013), and DMAT (Vandekerckhove & Tuerlinckx, 2008) has made the diffusion model easier to fit to data than was formerly the case and has increased its attraction for both basic and applied researchers as a result.

In response to the increased use of the diffusion model in a progressively wider range of settings, an increasing amount of attention has been paid to the validity of its estimated parameters. This has led to a literature of *selective influence* studies. Historically, the term “selective influence” dates from Sternberg’s (1969) additive-factors study of stage models, where it referred to the assumption that an experimental manipulation should affect only one of a hypothesized sequence of processing stages. In recent model-based studies it has instead been used to express the requirement that an experimental manipulation should affect the model parameter it is theoretically predicted to affect and no other (Jones & Dzhafarov, 2014). Selective influence can be characterized in a wholly abstract way, as conditional independence among members of a set of random variables, given the values of experimental factors that affect the members of the set (Dzhafarov, 2003), but we use the term here in Jones and Dzhafarov’s more informal, model-based sense.

In the diffusion model, there are four parameters that lead to clear selective influence predictions. These are the drift rate, *ν*, the boundary separation, *a*, the starting point for evidence accumulation, *z*, and the nondecision time, *T*_er_. The drift rate characterizes the quality of the information in the stimulus; the boundary separation characterizes the amount of evidence needed for a response; the starting point characterizes the response bias, and the nondecision time characterizes the time for other, nondecision (“encoding and response”) processes. Experimentally, we would expect drift rate to be affected by stimulus discriminability, boundary separation to be affected by speed-accuracy instructions, starting point to be affected by prior probabilities and payoffs, and nondecision time to be affected by any manipulation that changes overall processing time without changing either the quality of the evidence in the stimulus or the amount of evidence needed for a response. Shwartz et al., (1977) used the additive-factors method to show that stimulus luminance affects stimulus encoding and stimulus-response compatibility affects response selection in two-choice response time (RT). In the diffusion model, the times for stimulus encoding, response selection, and response execution together comprise the nondecision time.

Selective influence studies have produced mixed results. While many studies have imposed a priori selective influence constraints and obtained excellent fits to data, there is a body of anomalous findings from studies that have not constrained the model parameters but have allowed them to vary freely. For example, manipulations of speed-accuracy settings have been found to affect both nondecision times (Arnold et al., 2015; de Hollander et al., 2016; Donkin et al., 2011; Huang et al., 2015) and drift rate variability (Heathcote and Love, 2012). Fontanesi et al., (2019) reported that nondecision times in a value-based decision task were affected by decision frames and prior information. The most challenging of these findings is that speed-emphasis instructions can lead to decreased estimates of drift rates (Donkin et al., 2011; Heathcote and Love, 2012; Ho et al., 2012; Rae et al., 2014; Starns et al., 2012; Vandekerckhove et al., 2008). That is, reducing the amount of evidence needed for a response seems to decrease the quality of the evidence extracted from the stimulus. Although it is possible to rationalize these violations of selective influence, they have no natural interpretation within the semantics of the model.

## The blinded validity study of Dutilh et al. (2019)

In response to these validity concerns, Dutilh et al., (2019) recently reported a large-scale, blinded parameter recovery study, involving 17 teams of researchers, each of whom tried to infer the manipulation(s) responsible for the experimental effect in 14 two-condition sets of RT and accuracy data. The decision task was the random dot motion (RDM) task, in which the decision maker attempts to identify the direction of coherent motion in random dot kinematograms. The RDM task was originally developed as a pure motion task, in which a global motion signal must be extracted from the ensemble statistics of an array of local motion vectors in the absence of systematic displacement cues from which direction of motion can be inferred (Baker and Braddick, 1982; Newsome & Paré, 1988; van de Grind et al., 1983). It was repurposed to study decision making, initially in awake, behaving monkeys (Shadlen & Newsome, 2001) and later in humans (Palmer, Huk, & Shadlen, 2005).

Dutilh et al., (2019) studied performance in the RDM task in a 2 × 3 × 2 (Speed-Accuracy × Bias × Discriminability) experimental design. Speed-accuracy settings were manipulated by instructions; bias was manipulated by the relative frequencies of the two stimuli within an experimental block, and discriminability was manipulated by varying the coherence of the motion. (Dutilh et al. termed the speed-accuracy factor “caution” and the discriminability factor “ease.”) From this design, they created 14 different two-condition data sets in which zero, one, two, or three experimental variables differed between the two conditions. The challenge for the participating researchers was to infer which variable or variables differed between conditions on the basis of the RT and accuracy data alone.

The 17 teams used a diverse range of models and methods. They used several variants of the diffusion model, ranging from the simple (Wagenmakers et al., 2007) to the complex (Ratcliff and McKoon, 2008), the linear ballistic accumulator (LBA; Brown & Heathcote, 2008), and informal “chi by eye” inference from the qualitative changes in the RT distributions and accuracy statistics. They used a variety of fitting methods, both classical and Bayesian, hierarchical and nonhierarchical, to fit the models to data. Although there were common method variance effects, in which teams that used similar methods and models tended to obtain similar results (Dutilh et al.,, 2019, Figure 2), the similarities greatly outweighed the differences. Overall, the diffusion model performed slightly better than the LBA, but both models were generally successful in correctly identifying the manipulated variables in each of the 14 data sets, consistent with previous reports that the diffusion model and the LBA often make very similar predictions (Donkin et al., 2011).

The most striking and puzzling result was the high proportion of false alarms (misidentified effects) involving nondecision times. None of the three experimental variables manipulated in the study were intended to affect nondecision time, but an appreciable number of the researchers, as well as correctly identifying the variable that had changed, incorrectly inferred that nondecision time had also changed (Dutilh et al.,, 2019, Figure 3). The authors commented that the majority of these false alarms came from the diffusion model. For the full diffusion model, the overall accuracy of parameter recovery was 73%, but this went up, in the best case (Starns, minimum Chi-square, individual participant fits), to 93% once false alarms involving nondecision time were discounted. These findings replicate those of de Hollander et al., (2016) and Huang et al., (2015) who also found that estimates of nondecision time in the diffusion model were affected by speed-accuracy instructions in the RDM task. In addition to the false alarms involving nondecision times, there was also a tendency for both diffusion and LBA models to incorrectly misattribute manipulations of caution (speed vs. accuracy instructions) to a combination of caution and stimulus discriminability, echoing the findings of earlier studies.

How are we to interpret these failures of selective influence? The glass-half-full interpretation is that the models correctly identified the variables associated with the differences between conditions in many cases—although this positive result is qualified by the good performance of the “chi by eye” teams who often correctly identified the manipulation without recourse to any kind of model-based inference. The glass-half-empty interpretation is that selective influence, in the strictest sense, was comprehensively violated. One can attempt to retrieve the situation, as the authors did, by arguing that the true state of nature is that manipulations of speed-accuracy settings do indeed affect nondecision times. It is certainly plausible that people attempting to go fast maintain an elevated level of tonic activity in their effector muscles to facilitate recruitment of motor units and this may appear as a nondecision time effect in model fits. In support of this view, Dutilh et al. cited an electrophysiological study by Rinkenauer et al., (2004) using lateralized readiness potentials that suggested that speed-accuracy settings affect nondecision times. However, this explanation does not account for the finding of Dutilh et al. and earlier studies that manipulations of speed-accuracy affect both boundaries and drift rates.

In this article, we present evidence for a different point of view. We argue that the RDM task has unusually long temporal integration characteristics that may not be well captured by models in which the onset of evidence accumulation is abrupt. Both the diffusion model and the LBA model assume that evidence accumulation begins abruptly after a random onset time. In the diffusion model, there are two parameters, the drift rate and the diffusion rate (the so-called “diffusion coefficient”), that jointly control evidence accumulation. The former controls the rate of evidence accumulation; the latter controls how noisy it is. Mathematically, the drift rate and the diffusion coefficient are modeled as random step functions: At some random time, typically, on average, between 300 and 600 ms after stimulus onset (Matzke & Wagenmakers, 2009, Figure A1), drift and diffusion rates go from zero to constant, nonzero values, *ν*, and *s*^2^, respectively.^1^ If stimulus encoding really is rapid relative to the time scale of the decision process, then the abrupt-onset assumption should be able to capture its dynamics fairly well—particularly if the onset time is allowed to vary randomly across trials. Estimates of this variability can range from 0 ms to more than 350 ms, with a mode of around 150-250 ms (Matzke & Wagenmakers, 2009, Figure A1). If, on the other hand, encoding is extended in time, so that the instantaneous evidence entering the decision process increases progressively over several hundred milliseconds, then the abrupt-onset assumption may have difficulty in capturing its dynamics. Our hypothesis is that this difficulty will manifest itself as a failure of selective influence, particularly with regard to drift rates and nondecision times. Dutilh et al. offered no strong reasons for choosing the RDM task other than to note “it is a popular task, and we hope our results can be reasonably generalized to other simple decision-making tasks” (Dutilh et al.,, 2019, p. 1056). Our study was motivated by our reservations about this latter claim.

## The psychophysics of visual temporal sensitivity

There are multiple stages of integration that may intervene between the presentation of a stimulus and the production of a response. Minimally, the process of forming a perceptual representation of a stimulus involves one stage of integration and the process of accumulating noisy samples of that representation to make a decision involves another. These two stages may operate sequentially in a strict, stage-dependent way, as envisaged in the additive-factors model of Sternberg (1969), or they may overlap in time, as envisaged in the cascade model of McClelland (1979). The classical literature on visual temporal sensitivity developed methods to study perceptual integration experimentally and to model it mathematically (Watson, 1986). The most direct way to characterize perceptual integration is via threshold-versus-duration (TvD) functions, which plot discrimination “thresholds” (the level of stimulus intensity or discriminability needed to produce a criterion level of accuracy), as a function of stimulus duration, which is systematically varied.

**Fig. 1:**
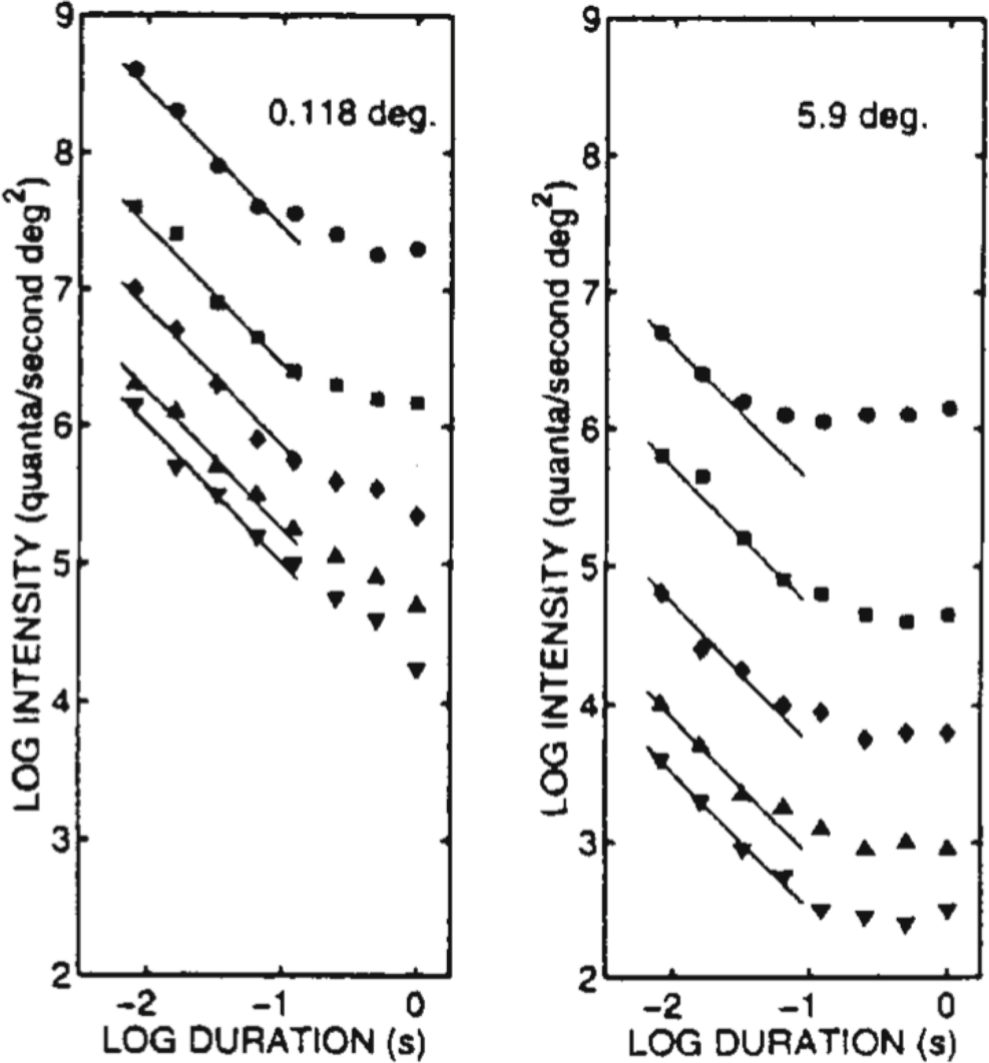
Threshold-versus-duration functions for detecting small (0.118^∘^) and large (5.9^∘^) luminous disks. The individual functions in each figure are for decreasing levels of background luminance (*top to bottom*). The *straight lines* in each figure have slopes of -1 (Bloch’s law) and extend to an exposure duration of 100 ms. Redrawn from Barlow (1958). “Temporal and spatial summation in human vision at different background intensities,” *Journal of Physiology, 141,* pp. 337–350. Reprinted from *The Australian Journal of Psychology, 50,* P. L. Smith, “Bloch’s law predictions from diffusion process models of detection,” 139–147, 1998, Fig. 1. Copyright the Australian Psychological Society

The most basic expression of temporal integration in the early visual system is Bloch’s law (Bloch, 1885; Gorea, 2015), which says that for short stimulus durations, up to a critical duration, *d*_*c*_, the visual system functions as a perfect temporal integrator. More formally, if *I* denotes stimulus intensity and *d* denotes duration, Bloch’s law states that *Id* = *c* (constant) for *d* ≤ *d*_*c*_. For many stimuli, *d*_*c*_ is of the order of 80-100 ms. Figure 1 reproduces some classic data from Barlow (1958) on detecting small and large luminous disks on uniform backgrounds of varying intensities. The data are plotted in log-log coordinates, so Bloch’s law appears as a straight line with slope of -1, as shown in the figure. For our purposes, the most important feature of these data is that in most conditions there is a fairly clear transition from the Bloch’s law regime, in which thresholds decrease linearly, to longer durations, in which thresholds decrease more slowly, or do not decrease at all. Similar results for detecting sinusoidal grating stimuli were reported by Breitmeyer and Ganz (1977) (see also Gorea & Tyler, 1986, Figure 1). Less important for us, although fundamental to theories of visual temporal sensitivity, is the differential breakdown of Bloch’s law at long durations for large and small stimuli. For large disks, thresholds show no further reduction beyond the Bloch’s law regime; for small disks, they continue to decrease but at a slower rate. The reduction in this part of the function can often be described by a straight line with slope − 1/2, which represents a square-root law. The square-root law regime has been interpreted as indicating statistical integration of stimulus information by a decision process, as distinct from neural integration by the perceptual system in the Bloch’s law regime (Watson, 1979). Smith (1998, Figure 4) showed that the contrasting patterns of threshold reduction in Fig. 1 can be well described by the diffusion model of Smith (1995) in which an Ornstein-Uhlenbeck diffusion decision process is driven by linear filters, which represent the outputs of sustained and transient perceptual channels (Breitmeyer, 1984).

**Fig. 2:**
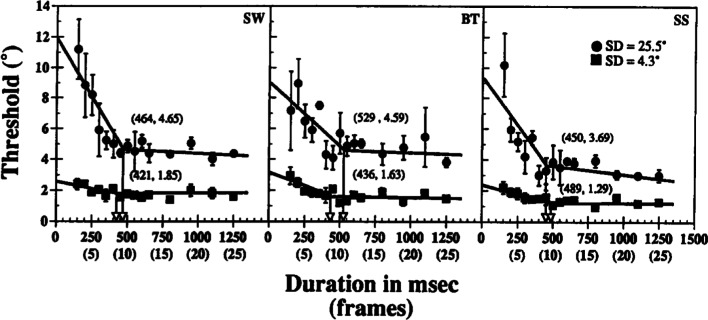
Threshold-versus-duration functions for discriminating direction of motion in random dot kinematograms. Thresholds were measured using an adaptive staircase procedure that identified the level of motion coherence needed to produce 84% discrimination accuracy for a given exposure duration. The *circles* and *squares* are the measured thresholds for high (*SD* = 4.3^∘^) and low (*SD* = 25.5^∘^) coherence stimuli, respectively. The data were fit with bilinear functions to identify the limits of temporal integration. The end points of the periods of temporal integration for the two levels of motion coherence are marked by *arrows* projecting from the knee of the function to the time axis. The *figures in parentheses* are the means and standard errors of the estimated temporal integration time. Reprinted from *Vision Research, 32,* S. N. J. Watamaniuk & R. Sekuler, “Temporal and spatial integration in dynamic random dot stimuli,” 2341–2347, 1992, Fig. 2, with permission from Elsevier

Figure 2 shows the results of a temporal integration study of the random dot motion task by Watamaniuk and Sekuler (1992). Three observers performed the task at two different levels of motion coherence, which was manipulated by varying the standard deviation of the dot motion. The data were fit with a bilinear function whose knee identifies the critical duration for temporal integration. Two features of Fig. 2 are striking. First, the period of temporal integration is much longer than in Fig. 1. Unlike Fig. 1, in which the critical durations are 100 ms or less, the critical durations in Fig. 2, which are fairly similar for the three observers, have a mean of around 450 ms. Second, the critical durations are very similar for high and low coherence stimuli. In Fig. 1, there is a natural identification of the two limbs of the TvD function with the perceptual and decision-making components of processing in an evidence-accumulation model. If this identification is correct, then it implies that the processes that give rise to drift rates for detecting spots of light can be completed in under 100 ms. However, it is much more difficult to know how to identify the components of the model with the curves in Fig. 2.

Watamaniuk (1993) showed that the reduction in thresholds over the first 400 ms in the RDM task follows a square-root law—but how this reduction should be interpreted is not clear. One interpretation is that it represents evidence accumulation by a decision process (Palmer et al., 2005). But if so, then it seems to imply that drift rates can be computed virtually instantaneously, with no initial Bloch’s law regime during which a perceptual representation is formed before the onset of the decision process. The other interpretation is that the decreasing limb of the function represents the perceptual processes that give rise to drift rates. If drift rates depend on the ensemble statistics of local motion vectors via some kind of averaging process, then it is plausible that they will increase more slowly with duration than do similar computations for spots of light and grating patches. Under this interpretation, the 400-450 ms critical duration represents the temporal integration limit of the global motion system, beyond the critical duration, the quality of the perceptual representation of motion will show no further improvement. The strongest argument for identifying the 400-450 ms period of threshold reduction in Fig. 2 with perceptual rather than decisional integration is that it is strikingly stable across observers and coherence conditions. This kind of invariance is what we might expect from a hard-wired, perceptual integration process, whereas if it were decisional, and hence subject to strategic control, then we might expect it be more variable, both across individuals and across conditions.

The interpretation of Fig. 2 is made more difficult by the fact that not all studies have shown such clear evidence of a constant critical duration as that of Watamaniuk and Sekuler (1992). Watamaniuk et al., (1989) and Williams and Sekuler (1984) obtained similar estimates to theirs, but Burr and Santoro (2001) obtained estimates of around 1000 ms. Gold and Shadlen (2003) reported a square-root law reduction in thresholds out to 750 ms, although their data show a systematic reduction in the rate of threshold change at long exposures that does not appear well fit by the straight line they used to characterize it (their Figure 6C). Robertson et al., (2012) compared normal and autistic participants in the RDM task and found a 400 ms critical duration in normals but no evidence of a critical duration in autistic participants. Some of the differences in the reported critical durations may be due to differences in the way stimuli were constructed and displayed. Scase et al., (1996) noted that different laboratories have used a variety of different methods for constructing RDM stimuli, which result in stimuli with different statistical characteristics. They found relatively small differences in the coherence thresholds measured using different methods, but they did not investigate whether there were any differences in the associated critical durations. These differences highlight the difficulty in unambiguously distinguishing the effects of stimulus duration on perceptual integration from its effects on decision-making.

One further piece of evidence that seems to support the idea of a critical duration of around 400 ms in the RDM task was provided by Holmes et al., (2016), who carried out a study in which the direction of motion changed unpredictably on some trials. They fit their data with a piecewise LBA model in which the drift rates had one value before a change and another after it. The best-fitting model was one in which the drift rates changed around 400 ms after the stimulus change. This is consistent with the idea that drift rates are the output of a perceptual integration process with an integration time of around 400 ms.

## A model with time-varying drift and diffusion rates

In this article, we refit the full data set from the Dutilh et al., (2019) study with several versions of the standard diffusion model, in which drift and diffusion rates are modeled as random step functions. We compare them to a model based on the integrated system model of Smith and Ratcliff (2009), in which drift and diffusion rates increase progressively over time. Smith et al., (2014) showed that a version of this latter model provided a good account of performance in a related task, that of detecting pairs of stimuli (letters, bars, and grating patches) embedded in dynamic noise (Ratcliff and Smith, 2010). Like the RDM task, the dynamic noise task involves an extended period of perceptual integration, in which stimuli appear to emerge progressively from the noise. The RT distributions and choice probabilities (accuracy) from this task cannot be fit by the standard diffusion model unless nondecision times are allowed to vary with noise level (Ratcliff & Smith, 2010), but they are well fit by a model with constant nondecision times and drift and diffusion rates that increase over time. The success of the model in accounting for performance in the dynamic noise task suggests it may be a plausible model for the RDM task—although the tasks are different in important ways. The dynamic noise task requires identification of static form embedded in noise whereas the RDM task requires extraction of a global motion signal from noise in the absence of form.

In addition to the processes of perceptual integration and evidence accumulation discussed above, the integrated system model includes several component submodels. There is a further stage of integration that characterizes the formation of a visual short-term memory trace, a spatial attention stage that gates the evidence accumulation by the decision process, and a model of the time course of visual masking. The submodels are all governed by smooth temporal dynamics and sequentially arranged processes (perception, memory, and decision-making) operate in cascade. The submodels allow the model to account for a variety of attention, memory, and masking findings and the interactions among them (Gould et al., 2007; Ratcliff & Rouder, 2000; Sewell & Smith, 2012; Smith et al., 2014; Smith et al., 2010; Smith et al., 2004). However, the design of the Dutilh et al., (2019) experiment, which used centrally presented, response-terminated stimuli, is not suitable for fitting the full integrated system model. Instead, we considered a restricted form of the model that abstracts out its essential properties, namely, that drift and diffusion rates increase smoothly and progressively over time to an asymptote. Our motivation for considering the restricted model was to test the hypothesis that drift and diffusion rates vary over time using the fewest possible parameters.

The models we consider here assume that evidence accumulation in the decision process is governed by some version of the time-dependent stochastic differential equation^2^

1 $$ dX_{t} = \mu(t)  dt + \sigma(t) dW_{t}.  $$

In this equation, *dX*_*t*_ is the random change in evidence in the decision process during a small interval of duration *dt*, *μ*(*t*) is the drift rate, *σ*(*t*) is the infinitesimal standard deviation, and *dW*_*t*_ is the random change in a standard Wiener, or Brownian motion, diffusion process during the interval *dt*. As in the standard model, the drift rate controls the rate at which evidence accumulates and the infinitesimal standard deviation controls its noisiness, but here they are both modeled as time-dependent functions. The square of the infinitesimal standard deviation, *σ*^2^(*t*), is the diffusion coefficient. Diffusion processes like the one in Eq. 1, in which drift rates and/or diffusion rates change over time are referred to as *time-inhomogeneous* processes. This contrasts with the standard diffusion model, in which the drift and diffusion rates are constant within a trial: Such processes are termed *time-homogeneous*. (The Wiener diffusion process is also spatially homogeneous. This means it can be translated in evidence space, simply by relabeling the boundaries and starting point, without changing any of its properties.)

A model with well-behaved properties can be obtained if *μ*(*t*) and *σ*^2^(*t*) both grow in proportion to a common time base, *𝜃*(*t*), where the latter is some smooth function of time, so that

2 $$ dX_{t} = \mu \theta(t)  dt + \sigma \sqrt{\theta(t)} dW_{t}.  $$

Smith et al., (2014) called this model a *time-changed* diffusion because it can be obtained from the standard model by a change in its time scale.

It is of interest to consider a slightly more general model than this, in which there are two sources of diffusion noise, one which is dependent on the stimulus and another that is independent of it. In this generalized form of the model, evidence accumulation is governed by the equation,

3 $$ dX_{t} = \mu \theta(t)  dt + \sigma_{1} \sqrt{\theta(t)} dW_{t}^{(1)} + \sigma_{2}  dW_{t}^{(2)},  $$

where $W_{t}^{(1)}$ and $W_{t}^{(2)}$ are independent Brownian motions. By the additive property of Brownian motion, this model can equivalently be viewed as a process with a single coactive source of noise, *W*_*t*_, with infinitesimal standard deviation $\sigma (t) = \sqrt {{\sigma _{1}^{2}}\theta (t) + {\sigma _{2}^{2}}}$, after a suitable rescaling of coefficients.^3^ The evidence accumulation equation can therefore be written more simply as

4 $$ dX_{t} = \mu \theta(t)  dt + \sqrt{{\sigma_{1}^{2}}\theta(t) + {\sigma_{2}^{2}}} dW_{t}.  $$

Following the scaling assumptions commonly made in the literature, we set *σ*_1_ = 0.1 and estimate *σ*_2_ from data. We refer to the function *𝜃*(*t*) as the *evidence growth* function. This terminology refers to the evidence entering the decision process, not to the accumulating evidence represented by the process *X*_*t*_. The latter grows regardless of whether drift and diffusion rates are constant or time-varying.

Our reason for considering the more general model of Eq. 4 is that it provides a plausible, alternative way to predict fast errors. In the standard model, fast errors are predicted by variability in the starting point for evidence accumulation, *z*. In the model of Eq. 4, if the function *𝜃*(*t*) grows smoothly from zero at *t* = 0, then evidence accumulation early in a trial, when the drift rate is near-zero, will be dominated by the constant noise term, which will make early crossings of the wrong boundary more likely, leading to fast errors. Smith and Ratcliff (2009) showed that such a combination of constant and time-varying noise allowed the integrated system model to predict the fast errors in a data set reported by Gould et al., (2007) in which low contrast grating patches were presented on a uniform field. Smith et al., (2014) showed the same mechanism allowed the model to predict the fast errors in the dynamic noise task reported by Ratcliff and Smith (2010). The second source of diffusion noise can be thought of as characterizing a tendency for the decision-maker to sample noise from the display in the absence of stimulus information, as originally proposed by Laming (1968). Following him, we refer to this source of noise as “premature sampling noise.”

As in the standard model, the predictions of the time-varying model are obtained from the first-passage time distributions of the evidence accumulation process through the decision boundaries. When the drift and diffusion rates are constant, these predictions can be obtained from an infinite series representation (Cox and Miller, 1965; Ratcliff, 1978; Smith, 1990), but such representations do not exist for processes with arbitrary time-varying drift and diffusion rates. Instead, predictions can be obtained from integral-equation representations that can be discretized and solved recursively. The integral equation method was first proposed by Durbin (1971) and later developed to study the properties of integrate-and-fire neurons by Ricciardi and colleagues (Buonocore, Nobile, & Ricciardi, 1987; Buonocore, Giorno, Nobile, & Ricciardi, 1990). A pioneering study by Heath (1992) used Durbin’s method to study the cascade model of McClelland (1979). A detailed account of these methods can be found in Smith (2000).

The quantities of interest for predicting RT distributions and accuracy are the joint first-passage time densities for the process through the upper and lower boundaries, which we denote *g*_*A*_(*a*_1_,*t*|*z*,0) and *g*_*B*_(*a*_2_,*t*|*z*,0), respectively. The conditional notation expresses the idea that these functions are first-passage time densities for a process *X*_*t*_ starting at *z* at time 0, *X*_0_ = *z*, which makes a first boundary crossing at either *a*_1_ or *a*_2_ at time *t*. For a Wiener diffusion process starting at *z* at time zero, with decision boundaries *a*_1_ and *a*_2_, such that *a*_2_ < *z* < *a*_1_, the first-passage time densities for responses at the upper and lower boundaries have the integral equation representations

5 $$ \begin{array}{@{}rcl@{}} g_{A}(a_{1},  t|  z,  0) & = & -2{\Psi}(a_{1},  t|  z,  0) \\ & & + 2{{\int}_{0}^{t}} g_{A}(a_{1},  \tau|  z,  0) {\Psi}(a_{1},  t|  a_{1},  \tau) d\tau \\ && + 2{{\int}_{0}^{t}} g_{B}(a_{2},  \tau|  z,  0) {\Psi}(a_{1},  t|  a_{2},  \tau) d\tau \end{array} $$

6 $$ \begin{array}{@{}rcl@{}} g_{B}(a_{2},  t|  z,  0) & = & 2{\Psi}(a_{2},  t|  z,  0) \\ && - 2{{\int}_{0}^{t}} g_{A}(a_{1}, \tau|  z,  0) {\Psi}(a_{2},  t|  a_{1},  \tau) d\tau \\ && - 2{{\int}_{0}^{t}} g_{B}(a_{2},  \tau|  z,  0) {\Psi}(a_{2},  t|  a_{2},  \tau) d\tau. \end{array} $$

The first-passage time densities in Eqs. 5 and 6 are defined as the integrals of the products of their values at times *τ* < *t* and of a kernel function Ψ(*a*_*i*_,*t*|*a*_*j*_,*τ*), *i*,*j* = 1,2, which depends on the boundaries *a*_1_ and *a*_2_ and on the transition density of a time-varying Wiener process that satisfies Eq. 4. In Appendix A it is shown that the kernel function for Eq. 4 has the form

7 $$ \begin{array}{@{}rcl@{}} {\Psi}(a_{i},  t|  a_{j},  \tau) & = & \frac{1}{\sqrt{2\pi[ {\sigma_{1}^{2}}{\int}_{\tau}^{t} \theta(s) ds + {\sigma_{2}^{2}}(t - \tau)]}}\exp\left\{-\frac{\left[a_{i} - a_{j} - \mu {\int}_{\tau}^{t} \theta(s) ds\right]^{2}} {2[{\sigma_{1}^{2}}{\int}_{\tau}^{t} \theta(s) ds + {\sigma_{2}^{2}}(t - \tau)]}\right\} \\ && \times \frac{1}{2}\left\{-\mu\theta(t) - \frac{a_{i} - a_{j} - \mu {\int}_{\tau}^{t} \theta(s)  ds} {[{\sigma_{1}^{2}}{\int}_{\tau}^{t} \theta(s) ds + {\sigma_{2}^{2}}(t - \tau) ]}[{\sigma_{1}^{2}}\theta(t) + {\sigma_{2}^{2}}]\right\}. \end{array} $$

The kernel function Ψ(*a*_*i*_,*t*|*a*_*j*_,*τ*) goes to zero as $\tau \rightarrow t$, which is a requirement for the representations of the first-passage time densities in Eqs. 5 and 6 to be numerically stable (Buonocore et al., 1987).

Equation 7 may be compared to the kernel functions for a Wiener diffusion process with time-varying drift rate and constant diffusion rate given by Smith (2000; Equation 57) and for a process with time-varying drift and diffusion rates given by Smith et al. (2014; Equations B8 and B9). The kernel in Eq. 7 is more complex than in either of those applications because of the presence of two diffusion terms in Eq. 4, one of which is time-varying and one of which is not. In applications, the solutions in Eqs. 5 and 6 are evaluated numerically by defining the process on a discrete time mesh, *t*_*i*_ = *i*Δ, *i* = 0,1,2,…, and approximating the integrals with discrete sums. The discretized forms of the equations can be found in several places including Smith (2000; Equations 47a and 47b) and Voskuilen, Smith, and Ratcliff (2016; Appendix B), and are reproduced in Appendix A here.

The integral equation method is sufficiently general and flexible that it can also be used to obtain predictions for models with time-varying boundaries, *a*_1_(*t*) and *a*_2_(*t*). Voskuilen et al. (2016, Appendix B) give the kernel function for a Wiener process with fixed drift and diffusion rates and time-varying boundaries. This representation provides an explicit mathematical method for studying the so-called “collapsing boundary problem” (Hawkins et al., 2015), as we discuss subsequently.

In the integrated system model of Smith and Ratcliff (2009), the function we denote here as *𝜃*(*t*), which controls the growth of the drift and diffusion rates, depends on the output of perceptual and visual short-term memory processes acting in cascade. The dynamics of the cascade depend on three different rate constants that control the rate of perceptual processing by early visual filters, the decay of the perceptual representation after stimulus offset or its suppression by masks, and the rate of visual short-term memory formation. The model has similar temporal dynamics to those in the visual short-term memory model of Loftus and colleagues (Busey & Loftus, 1994; Loftus & Ruthruff, 1994), but, unlike their model, the strength of the visual short-term memory trace determines the drift and diffusion rates of a diffusion process. Here, instead of fitting the full model, we assumed that the growth-rate function *𝜃*(*t*) had the form of an *n*-stage cumulative gamma function, with rate parameter *β* of the form

8 $$ \theta(t) = 1 - \sum\limits_{i=0}^{n-1} \frac{(\beta t)^{i}}{i!} e^{-\beta t}.  $$

When viewed as a deterministic function rather than as a probability distribution, the cumulative gamma describes the output of a linear system composed of a cascade of *n* exponential (RC or “resistance-capacitance”) stages. There is a long tradition in visual psychophysics, dating back to the pioneering work of de Lange (1958), of using linear-system theory to represent the visual temporal response function (Smith, 1995; Sperling and Sondhi, 1968; Watson, 1986). The representation of Eq. 8 therefore connects to this classical literature on visual temporal sensitivity. In addition, Eq. 8 satisfies the smoothness requirements of the integral equation method, which requires that functions in the kernel be at least twice differentiable.

The discreteness of the parameter *n* in Eq. 8 is inconvenient when fitting models to data, so we implemented our models using the incomplete gamma function, which is a continuous-parameter generalization of Eq. 8. Keeping the same notation, the incomplete gamma function has the form (Abramowitz & Stegun, 1965; p, 260, Equation 6.5.1)

9 $$ \theta(t) = \frac{1}{{\Gamma}(n)}{\int}_{0}^{\beta t} e^{-s} s^{n-1} ds,  $$

where Γ(*n*) is the gamma function, which coincides with the factorial function, Γ(*n*) = (*n* − 1)!, when *n* is an integer. For integer *n*, Eqs. 8 and 9 are equal. The integral in Eq. 9 does not have a closed-form solution but efficient routines for evaluating it numerically can be found in most libraries of special functions. Together, the first-passage time densities of Eqs. 5 and 6, the kernel function of Eq. 7, and the evidence growth function of Eq. 9 provide sufficient mathematical structure to fully constrain the model. The important parameters in fitting the model to data are the rate and shape parameters, *β* and *n*, which control the time course of the evidence entering the decision process, and the constant source of diffusion noise, *σ*_2_, which controls the model’s propensity to predict fast errors. Although the time-varying model has three parameters that the standard model does not, we found it could fit the Dutilh et al., (2019) data without across-trial variability in either starting point or nondecision time. This resulted in models with exactly the same number of free parameters, as we discuss below.

## Method

The data from the Dutilh et al., (2019) study are publicly available and downloadable from the Open Science Foundation. The full data set comprises RT and accuracy data from 20 participants, each of whom completed around 2800 trials. Because the authors manipulated bias by varying the relative frequencies of leftward and rightward motion, the number of trials was not fully balanced across conditions. In order to camouflage the experimental manipulations from the researchers in the blinded study, the authors used a rather complex block structure, the full details of which are described in the original article. Unlike the researchers in the blinded study, who fit the data from 14 different pairs of experimental conditions, we fit the data from the full experimental design. Also, unlike those researchers, we did so in the knowledge of the manipulations in each of the experimental conditions and made judicious use of selective influence assumptions in order to constrain the models. Apart from these differences, we attempted to follow the authors’ treatment of data as closely as possible.

When stimulus identity (leftward or rightward motion) is also taken into account there were a total of 24 conditions in the Speed-Accuracy × Bias × Discriminability design in their study. Like the authors, we pooled data from leftward and rightward stimuli in corresponding conditions. So, for example, under biasing manipulations, we pooled the data from blocks in which leftward motion had low probability with blocks in which rightward motion had low probability. We then relabeled the stimuli and responses as low and high probability stimuli with their associated correct and error responses. This reduced the number of experimental conditions to 12. In the pooled data, bias was represented by three conditions, conditioned on stimulus identity: low probability stimuli, equal probability stimuli, and high probability stimuli. The authors also excluded trials on which the RT was shorter than 200 ms as fast guesses. They expressed reservations about the propriety of this exclusion in their report, but, as the fastest visual simple RTs are around 200 ms, their exclusion criterion seems not only reasonable but conservative.

Indeed, after carrying out a preliminary analysis of the data, we increased the fast-guess exclusion cutoff to 280 ms. When filtered at 200 ms, the original data showed a pronounced fast-error effect under speed instructions, which appeared as a large shift in the leading edges (the .1 quantiles) of the error distributions. This shift proved difficult to fit with across-trial variability in starting point in the standard diffusion model (see Results section for details), although the time-varying model was able to capture it. Ratcliff and McKoon (2008) obtained excellent fits of the standard model to data from the RDM task, but their data showed smaller effects of speed instructions on the 0.1 error distribution quantiles (their Figure 9). Increasing the cutoff to 280 ms improved the fits of the standard diffusion model.^4^ The effect on the time-varying model, which has another mechanism for predicting fast errors, was much less evident.

Contrary to the usual practice in psychophysical studies, in which stimuli are tailored to the sensitivities of individual participants (Smith & Little, 2018), Dutilh et al., (2019) used two fixed levels of stimulus discriminability (easy and hard) for all participants. As result, the performance of many of the participants was at ceiling in some conditions. Ten of the participants had missing error RT data in one or more conditions and a further five had insufficient data to compute the quantiles of some of the error RT distributions. For these participants we followed the procedure for treating missing data in Ratcliff and Childers (2015), described below. To fit the data, we minimized the likelihood-ratio Chi-square statistic (*G*^2^) for the response proportions in the bins formed by the .1, .3, .5, .7, and .9 RT quantiles for the distributions of correct and error responses (Ratcliff & Smith, 2004). When bins are formed in this way, there are a total of 12 bins (11 degrees of freedom) in each pair of joint distributions of correct responses and errors.

The resulting *G*^2^ statistic can be written as

$$ G^{2} = 2\sum\limits_{i=1}^{12} n_{i} \sum\limits_{j = 1}^{12} p_{ij} \log\left( \frac{p_{ij}}{\pi_{ij}}\right). $$

In this equation, *p*_*ij*_ and *π*_*ij*_ are, respectively, the observed and predicted probabilities (proportions) in the bins bounded by the quantiles, and “$\log $” is the natural logarithm. The inner summation over *j* extends over the 12 bins formed by each pair of joint distributions of correct responses and errors. The outer summation over *i* extends over the two speed-accuracy conditions, the three bias conditions, and the two discriminability conditions. The quantity *n*_*i*_ is the number of experimental trials in each condition (here ${\sum }_{i} n_{i} \approx 2800$). Fitting the data to joint distributions in this way takes into account the fits to RT and accuracy because the magnitude of *G*^2^ reflects how closely the predicted probability masses in the distributions of correct responses and errors agree with the corresponding observed masses. When there were fewer than five errors in a condition, bin boundaries based on error quantiles could not be computed. In these cases, if there were at least two responses, we computed medians and characterized the associated error distribution with two bins (above and below the median); otherwise we characterized the error distribution with either zero or one bin, depending on the number of error responses. All of the fits we report were to individual subject data, but we show plots of quantile-averaged group data and fits based on group-averaged parameter estimates as an economical way to represent some of the main qualitative features of the data as a whole.

There are, and will continue to be, differences in opinion in the modeling community about the best way to fit RT data. The variety of fitting methods used in the blinded validity study and summarized in Table 3 of (Dutilh et al., 2019) article highlights the extent of these differences. As noted above, the best parameter recovery, once selective influence violations associated with nondecision times were set aside, was obtained from minimum Chi-square fits to the individual participants’ data, which is similar to the method we used here. To compare models with different numbers of parameters, we used standard model selection methods based on the Akaike information criterion (AIC; Akaike, 1974) and the Bayesian information criterion (BIC; Schwarz, 1978). These fit statistics are derived from different theoretical principles (one classical and the other Bayesian), but we used them in the spirit in which they are typically used in the modeling literature, as penalized likelihood statistics that impose more or less severe penalties on the number of free parameters in a model (Voss et al., 2019). As is well known, the AIC tends to gravitate towards more complex models with increasing sample sizes more quickly than does the BIC (Kass & Raftery, 1995).

In other work from our laboratory (Corbett & Smith, 2020; Smith & Corbett, 2019), we have used modified versions of the AIC and BIC that correct for overdispersion, that is, for sources of variance in the data that are not represented in the likelihood equations of the model. Although this approach has useful properties, in the interests of making our methods as similar as possible to those commonly used in the RT literature we report AICs and BICs in their standard forms. We note, however, that the propensity for the AIC to gravitate towards more complex models will be increased in the presence of overdispersion. For binned data, the AIC and BIC may be written as

$$ \begin{array}{@{}rcl@{}} \text{AIC} & = & G^{2} + 2m \\ \text{BIC} & = & G^{2} + m\log(N), \end{array} $$

where *m* is the number of free parameters in the model and $N = {\sum }_{i} n_{i}$ is the total number of observations on which the fit statistic was based. To fit the models, we obtained a minimum *G*^2^ from 10 runs of the Nelder-Mead simplex algorithm (Nelder & Mead, 1965), using randomly-perturbed estimates from the preceding run as the starting point for the next run.

## Results

**Fig. 3:**
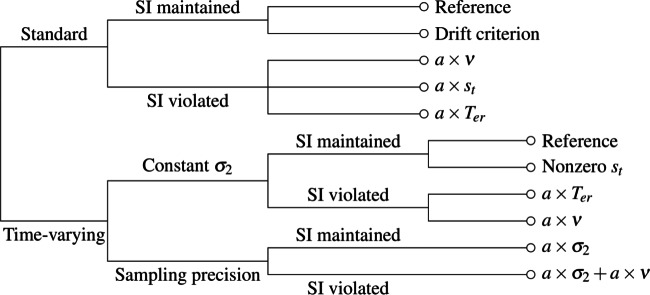
Relationships among models. In the standard models, drift and diffusion rates were constant within a trial; in the time-varying models, they varied with time. The “SI maintained” and “SI violated” models imposed and relaxed selective influence assumptions, respectively. The “SI violated” models are identified using an interaction notation described in the text. The sampling precision models allowed the premature sampling noise term, *σ*_2_, to vary with experimental instructions

We report fits of five versions of the standard diffusion model and four versions of the time-varying model, together with two extensions of the latter model. The full set of models and the relationships among them are summarized in Fig. 3. Some versions of the models were aimed at determining the best way to represent bias, particularly how best to characterize the fast errors found with speed-stress instructions. The remainder were aimed at characterizing violations in selective influence associated with nondecision times. Table 1 lists the parameters that were estimated in fitting the models to the data. The researchers in the Dutilh et al., (2019) study were free to parameterize the models in whichever way they thought was most appropriate and it is not clear from their article how the teams that used the standard diffusion model chose to parameterize it. Here we made selective influence assumptions that are typical of those found in the literature. The details of how we parameterized the models may be found in Appendix B. Although the time-varying model appears to have more free parameters than does the standard model, we were able to eliminate three of the standard model’s parameters when fitting the data, which made the number of free parameters in the two models exactly the same, as we discuss below.

**Table 1:** Parameters of the diffusion models

Parameter	Symbol	Level of effect
Boundary separation, speed	*a*(*s*)	block
Boundary separation, accuracy	*a*(*a*)	block
Mean drift rate, hard, speed^a^	*ν*(*hs*)	trial
Mean drift rate, easy, speed^a^	*ν*(*es*)	trial
Mean drift rate, hard, accuracy^a^	*ν*(*ha*)	trial
Mean drift rate, easy, accuracy^a^	*ν*(*ea*)	trial
Starting point bias	*π*_*z*_	block
Drift criterion	*c*_*ν*_	block
Drift rate variability	*η*	experiment
Starting point variability, speed	*s*_*z*_(*s*)	block
Starting point variability, accuracy	*s*_*z*_(*a*)	block
Mean nondecision time, speed^a^	*T*_er_(*s*)	experiment/block
Mean nondecision time, accuracy^a^	*T*_er_(*a*)	experiment/block
Nondecision time variability, speed^a^	*s*_*t*_(*s*)	experiment/block
Nondecision time variability, accuracy^a^	*s*_*t*_(*a*)	experiment/block
Evidence growth *𝜃*(*t*) rate^b^	*β*	experiment
Evidence growth *𝜃*(*t*) shape^b^	*n*	experiment
Premature sampling noise^b^	*σ*_2_	experiment

a= Varies with selective-influence assumptions; b = time-varying model only

### Standard diffusion models

Table 2 summarizes the parameters of the five versions of the standard diffusion model. Along with characterizing the effects of the experimental manipulations on drift rates and boundary settings, the models sought to identify violations of selective influence on nondecision times and drift rates. One model investigated whether the mean nondecision, *T*_er_, varied with boundary setting; another investigated whether nondecision time variability, *s*_*t*_, varied with boundary setting, and a third model investigated whether the mean drift rate, *ν*, varied with boundary setting. The selective influence violation models are identified in the tables using an interaction notation as *a* × *T*_er_, *a* × *s*_*t*_, and *a* × *ν*, respectively. The last model (Model 2 in the tables) investigated whether the effects of bias were better represented by a combination of drift rate bias, *c*_*ν*_, and starting point bias, *π*_*z*_, than by starting point bias alone (see Appendix B). We refer to the model with the usual selective influence assumptions, and which had the fewest free parameters, as the *reference model.* In all of our model fits, RTs were measured in units of seconds and the estimated parameters we report are for data scaled in this way, but in plots of fits to data we follow the convention in the literature of showing RTs in milliseconds.

**Table 2:** Standard diffusion models

Model	Properties	*m*	Parameters
1	Reference	10	*a*(*s*), *a*(*a*), *ν*(*h*), *ν*(*e*), *π*_*z*_, *η*, *s*_*z*_(*s*), *s*_*z*_(*a*), *T*_er_, *s*_*t*_
2	Drift Criterion	11	*a*(*s*), *a*(*a*), *ν*(*h*), *ν*(*e*), *c*_*ν*_, *π*_*z*_, *η*, *s*_*z*_(*s*), *s*_*z*_(*a*), *T*_er_, *s*_*t*_
3	*a* × *T*_er_	11	*a*(*s*), *a*(*a*), *ν*(*h*), *ν*(*e*), *π*_*z*_, *η*, *s*_*z*_(*s*), *s*_*z*_(*a*), *T*_er_(*s*), *T*_er_(*a*), *s*_*t*_
4	*a* × *s*_*t*_	11	*a*(*s*), *a*(*a*), *ν*(*h*), *ν*(*e*), *π*_*z*_, *η*, *s*_*z*_(*s*), *s*_*z*_(*a*), *T*_er_, *s*_*t*_(*s*), *s*_*t*_(*a*)
5	*a* × *ν*	12	*a*(*s*), *a*(*a*), *ν*(*hs*), *ν*(*ha*), *ν*(*es*), *ν*(*ea*), *π*_*z*_, *η*, *s*_*z*_(*s*), *s*_*z*_(*a*), *T*_er_, *s*_*t*_

*m* = number of free parameters

Table 3 summarizes the fits of the standard diffusion models. For all models, the *G*^2^, AIC, and BIC values in the table are averages for the 20 participants, as described in the Method section. The degrees of freedom are residual degrees of freedom for participants with no missing error data. The degrees of freedom for such participants are *df* = 12 × (12 − 1) − *m*, that is, the number of experimental conditions times the number of bins in each joint distribution pair minus one, minus the number of free parameters. For Models 2 through 5, the columns #AIC and #BIC are the numbers of participants for whom the model was preferred to the reference model, according to the AIC or the BIC. Table 4 gives the parameters of the best-fitting models, again averaged over participants.

**Table 3:** Fits of standard diffusion models

Model	Properties	*G*^2^	*df*	AIC	BIC	#AIC	#BIC
1	Reference	410.91	122	430.61	491.72	—	—
2	Drift Criterion	405.45	121	427.45	492.78	8	1
3	*a* × *T*_er_	392.84	121	414.84	480.17	12	11
4	*a* × *s*_*t*_	385.47	121	407.47	472.80	10	10
5	*a* × *ν*	390.16	120	414.16	485.43	11	8

Degrees of freedom unadjusted for individual missing data

**Table 4:** Parameters of standard diffusion models

Model	Properties	*a*(*s*)	*a*(*a*)	*ν*(*hs*)	*ν*(*ha*)	*ν*(*es*)	*ν*(*ea*)	*π*_*z*_	*c*_*v*_
1	Reference	0.083	0.150	0.165	—	0.297	—	0.058	—
2	Drift Criterion	0.083	0.150	0.168	—	0.302	—	0.050	-0.002
3	*a* × *T*_er_	0.086	0.146	0.164	—	0.296	—	0.053	—
4	*a* × *s*_*t*_	0.086	0.144	0.164	—	0.296	—	0.054	—
5	*a* × *ν*	0.085	0.156	0.153	0.174	0.272	0.316	0.056	—
		*η*	*s*_*z*_(*s*)	*s*_*z*_(*a*)	*T*_er_(*s*)	*T*_er_(*a*)	*s*_*t*_(*s*)	*s*_*t*_(*a*)	
1	Reference	0.055	0.023	0.004	0.258	—	0.247	—	
2	Drift Criterion	0.057	0.028	0.003	0.258	—	0.247	—	
3	*a* × *T*_er_	0.054	0.030	0.004	0.254	0.270	0.235	—	
4	*a* × *s*_*t*_	0.052	0.030	0.002	0.259	—	0.230	0.262	
5	*a* × *ν*	0.061	0.024	0.004	0.258	—	0.233	—	

For omitted entries, *ν*(*ha*) = *ν*(*hs*), *ν*(*ea*) = *ν*(*es*) *T*_er_(*a*) = *T*_er_(*s*) and *s*_*t*_(*a*) = *s*_*t*_(*s*)

The *G*^2^ statistics in Table 3 are comparable in magnitude to those reported previously from fits of the diffusion model to RDM data. The most relevant comparison study is that of Ratcliff and McKoon (2008) who fit the diffusion model to data from three experiments using the RDM task, each based on 960 trials per participant. Their first experiment varied motion coherence in six levels, their second crossed coherence with speed-accuracy instructions, and their third crossed coherence with the prior probability of leftward or rightward motion. The three tasks yielded Pearson *χ*^2^ fit statistics of 241, 421, and 723 from experimental designs with 55, 78, and 162 residual degrees of freedom respectively, resulting in *χ*^2^/*df* ratios of between 4.4 and 5.3. These ratios are several times their expected values under a central Chi-square sampling distribution, but graphically the fits to the three experiments appear excellent (their Figures 7, 9, and 10). The *G*^2^/*df* ratios for the models in Table 3 vary from around 3.2 to 3.5, which are comparable to those of Ratcliff and McKoon. Nonetheless, for reasons we discuss below, the Dutilh et al., (2019) data were challenging for the standard diffusion model to fit. We first discuss fits of the reference model and then consider the selective influence violation models.

#### Quantile-probability plots

**Fig. 4:**
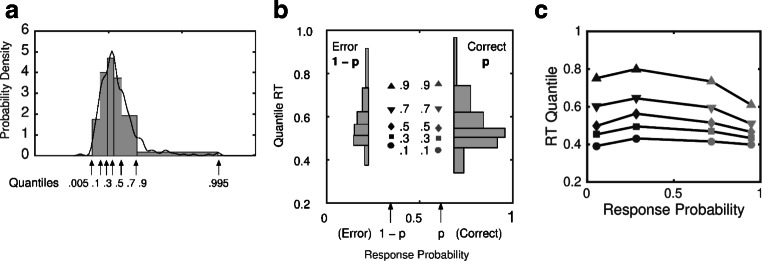
Constructing a quantile probability plot. **a** The RT distributions for correct responses and errors are summarized by histograms using the .1, .3, .5, .7, and .9 quantiles as bin boundaries. The continuous curve is a kernel density estimator applied to the same data. **b** For each stimulus condition, the quantiles of the distribution of correct responses are plotted against the probability of a correct response, *p*, and the quantiles of the distribution of error responses are plotted against the probability of an error response, 1 − *p*. **c** Quantile probability plot from an experiment with two stimulus conditions showing a slow-error pattern. Each condition contributes one pair of distributions to the plot

The most compact and effective way to represent the fit of a model to RT distributions and choice probabilities is in a quantile-probability plot. Figure 4 shows how such a plot is constructed from an experiment in which there are two discriminability levels, easy and difficult, like the Dutilh et al., (2019) study. To construct a quantile probability plot, the quantiles of the distribution of correct responses are plotted against the probability of a correct response, *p*, and the quantiles of the distribution of errors are plotted against the probability of an error response, 1 − *p*. Each stimulus condition contributes one pair of distributions to the plot. For an experiment like that of Dutilh et al., (2019) with two discriminability levels, there will be four distributions in the plot, like the example in Fig. 4. The distributions on the right side of the plot (light plotting symbols) are the distributions of correct responses and the distributions on the left side of the plot (dark plotting symbols) are distributions of errors. The innermost pair of distributions is from the difficult condition and the outermost pair is from the easy condition.

The plot shows how the RT distributions and choice probabilities vary as stimulus discriminability is changed. Most of the changes in RT with changing discriminability are in the upper quantiles of the distributions (the .5, .7, and .9 quantiles). The leading edge of the distribution (the .1 quantile) shows comparatively little change. The plot in Fig. 4 is canted upwards towards the upper left-hand side. This is the typical pattern of slow errors that is found in difficult tasks in which accuracy is stressed (e.g., Ratcliff & Smith, 2004). When there are fast errors the plot is canted downwards on the left-hand side. If there were no differences between the distributions of correct responses and errors, then the plot would be symmetrical across its vertical midline.

#### Reference model

**Fig. 5:**
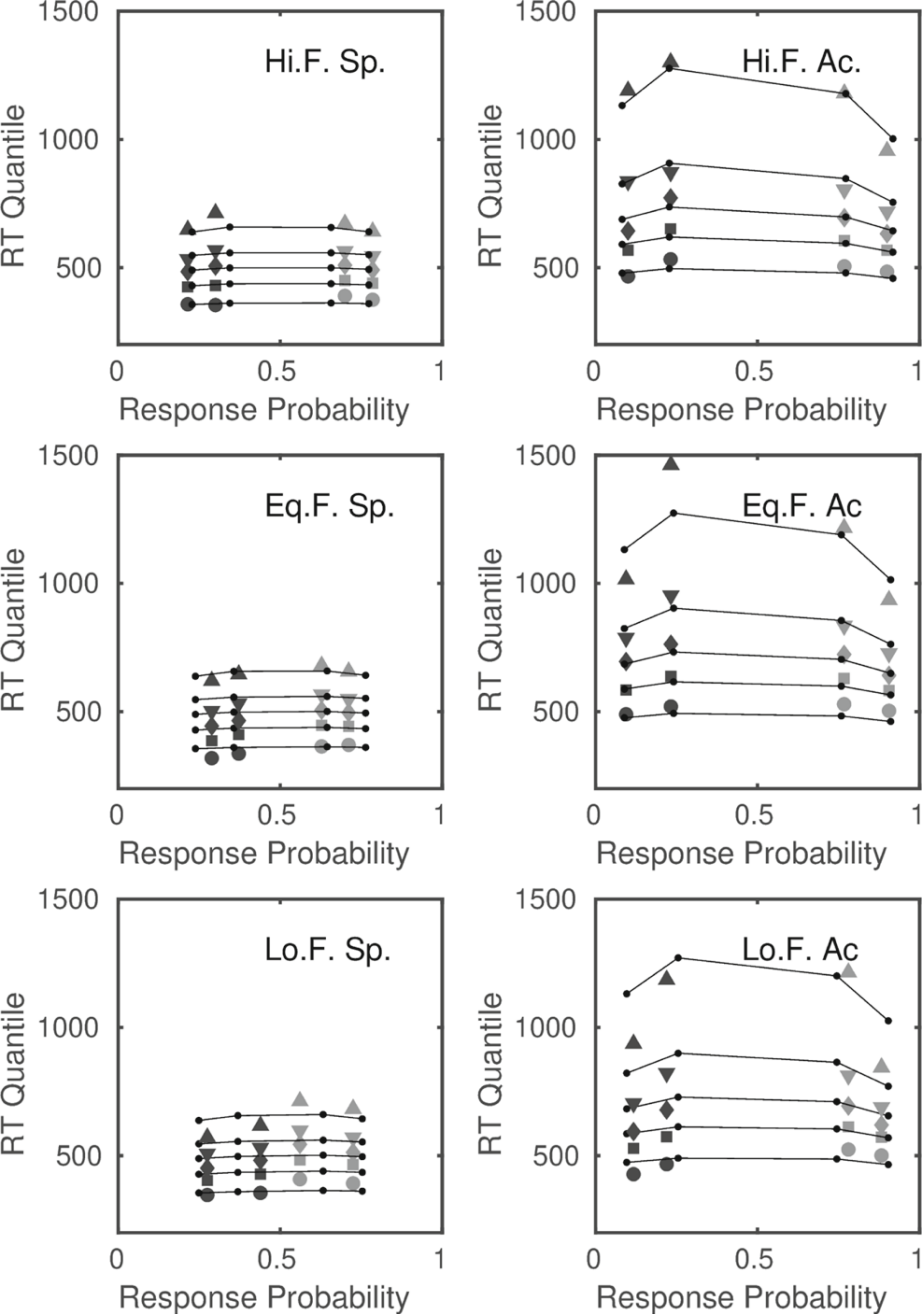
Quantile probability plot of the fit of the standard diffusion, reference model to participants with complete error data. The *columns* are speed (Sp.) and accuracy (Ac.) conditions. The *rows* are the bias conditions, conditioned on the stimulus. The *top and bottom rows* are high-frequency (Hi. F.) and low-frequency (Lo. F.) stimuli from blocks in which leftward and rightward motion was presented with unequal frequency. The *middle row* is from blocks in which leftward and rightward motion was presented with equal frequency (Eq. F.)

When individual differences are not too large, an effective way to represent the overall fit of a model is to use quantile-averaged group data (Ratcliff, 1979). To construct such a plot, corresponding quantiles of the distributions of correct responses and errors are averaged across participants, as are the choice probabilities. For the Dutilh et al. data, in which 15 participants were missing error distribution data in one or more conditions, we constructed the quantile probability plot from the data of the five participants (Participants 1, 6, 8, 11, and 19) for whom all distribution quantiles could be calculated. Figure 5 shows a quantile probability plot of the fit of the reference model (Model 1) to the quantile-averaged data for these participants. Although this plot represents only a subset of the full data, the main qualitative properties shown in the plot were replicated fairly consistently across the other participants, although with individual differences in RT and accuracy. In Fig. 5, the data are plotted conditioned on the stimulus (see figure caption). An alternative is to plot the data conditioned on the response (e.g., Ratcliff & McKoon, 2008).

Overall, the reference model captures the main features of the data, with two significant points of discrepancy. First, in the speed condition, the starting point bias parameter does not capture the variation in choice probabilities across high and low frequency stimuli, especially for the difficult stimuli. Second, and most challenging for the model, there is a pervasive fast-error pattern, which appears in both the speed and accuracy conditions. We discuss these effects in turn.

The estimated parameters in Table 4 show there was a small shift in the starting point towards the boundary associated with the more probable stimulus. This increased the probability that the associated response would be made, both correctly and incorrectly (i.e., correct responses in the top panels and error responses in the bottom panels). Conditioned on the stimulus, this translates into a greater difference in the choice probabilities for correct responses and errors (the horizontal extent of the plot) for high-frequency stimuli than for low-frequency stimuli. The model captures this difference in range fairly well for easy stimuli (the outermost pair of distributions in the plot) but not for difficult stimuli (the innermost pair). In contrast, in the accuracy condition, the model captures the choice probabilities for both easy and difficult stimuli well—although, as was noted by Dutilh et al., (2019) and is evident in the plot, the effect on the bias manipulation in the accuracy condition is comparatively small.

The joint effects of speed-accuracy instructions and stimulus prior probabilities is a fairly challenging pattern of data for models to explain. Ratcliff and McKoon (2008) studied both of these variables, and showed that the diffusion model accounted for them well, but not in the same experiment. In view of this additional constraint in the design, it was of interest to investigate whether the bias effects, when simultaneously manipulated with speed-accuracy settings, could be better accounted for by a combination of a drift criterion (Appendix B) and starting point changes. Model 2, which was identical to the reference model, except for the addition of a drift criterion, incorporated both of these effects.

The fit statistics in Table 3 show that the improvements obtained by adding a drift criterion, although discernible, were relatively small and inconsistent. The fits for eight of the participants were improved by the addition of a drift criterion according to the AIC, but for only one of them according to the more conservative BIC. The estimated mean value of the drift criterion, *c*_*ν*_ = − 0.002, implies that, on average, drift criterion affected drift rates only in the third decimal place, which translates into an almost negligible effect on choice probabilities. Because of the comparatively weak evidence for any effect of the biasing manipulation on the relative rates of evidence accumulation for high and low frequency stimuli, in what follows we treat the reference model as the baseline model for comparison with other models.

The most challenging feature for the models to explain was the systematic pattern of fast errors, which appears as a downward shift in the leading edge of the error distribution, relative to that of the distribution of correct responses, as measured by its .1 quantile. The pattern is apparent in Fig. 5, especially for equal-frequency and low-frequency stimuli in the speed condition. The shift in the .1 quantile is not confined to those participants for whom there was complete error distribution data: Averaging over all participants, bias conditions, and easy and difficult stimuli, the average .1 quantiles for correct and error responses in the speed condition were 383 ms and 330 ms, respectively, and in the accuracy condition 482 and 436 ms, respectively. In the accuracy condition, along with the downward shift in the .1 quantile, there is also an upward shift in the higher error distribution quantiles, shown schematically in Fig. 4c, which characterizes slow errors.

The combination of fast and slow errors is explained in the standard diffusion model by a combination of across-trial variability in drift rates and starting points (Ratcliff & Smith, 2004). Ratcliff and McKoon (2008) found evidence for both fast and slow errors in their experiments (e.g., their Figure 9) and successfully accounted for them using a combination of these two sources of variability. Our reference model also had a combination of drift rate variability and starting point variability, especially under speed instructions, but had difficulty in accounting for the shifts in the .1 quantiles of the error distributions. Indeed, our primary reason for changing the fast-guess cutoff from 200 ms to 280 ms was to try to improve this aspect of the fit. In comparison to Ratcliff and McKoon’s speed-accuracy experiment, participants in Dutilh’s et al.’s study were somewhat faster under speed instructions and had smaller boundary separations, which may have affected their propensity to make fast errors—although this was not reflected in the starting point parameters in Table 3, which are smaller than those reported by Ratcliff and McKoon. It is possible that the model misfits in Fig. 5 were due to the greater constraints imposed by Dutilh et al.’s experimental design, in which a biasing manipulation was crossed with a manipulation of speed versus accuracy. One consequence of combining starting point bias and starting point variability in the same model is that the values of the former restrict the permissible values of the latter: The more biased the starting point, the less it can vary and still remain within the boundaries. The comparatively poor fit of our reference model may reflect these constraints.

#### Selective influence violation models

Models 3 to 5 in Tables 3 and 4 are selective influence violation models. These models investigated whether mean nondecision time, *T*_er_, nondecision time variability, *s*_*t*_, and mean drift rate, *ν*, varied with speed versus accuracy instructions. Table 3 shows that the preferred model for many of the participants was one of the selective influence violation models. According to the AIC, model *a* × *T*_er_ was preferred to the reference model for 12 participants, model *a* × *s*_*t*_ was preferred to the reference model for 10 of them, and model *a* × *ν* was preferred to the reference model for 11. According to the more conservative BIC, *a* × *T*_er_ was preferred to the reference model for 11 participants, *a* × *s*_*t*_ was preferred for 10 of them, and *a* × *ν* was preferred for 8. There was little evidence of systematic effects across individual participants: It was not the case that the same subset of participants preferred all of the selective influence violation models over the reference model, suggesting that the models are reflecting different features of the individual data. Overall, one of the selective influence violation models was preferred to the standard model for 17 participants by the AIC and for 14 by the BIC.

The large proportion of selective influence violations involving nondecision times is in agreement with the findings of Dutilh et al., (2019), but, unlike them, we obtained these violations from the full experimental design. Dutilh’s researchers were set the challenging task of estimating model parameters from restricted, two-condition designs and it was not clear to us whether the selective influence violations they found were due to the inherent difficulties in obtaining stable estimates from minimal designs of this kind. The most systematic selective influence violation we found—in the sense of the one involving the most participants—was in *T*_er_, but there were also violations in *s*_*t*_ and *ν*. These violations are consistent with what has been reported previously in the literature. We conclude that the large number of selective influence violations reported by Dutilh’s researchers was not an artifact of the minimal designs from which they inferred the model parameters, but was, rather, a property of the data set as a whole. In the following section we report fits of a corresponding set of time-varying diffusion models.

### Time-varying diffusion models

Table 5 summarizes the time-varying models and their associated parameters. Like the standard models in Table 2, the set of models includes a reference model and selective influence violation models. As discussed earlier, the additional parameters in these models, *β*, *n*, and *σ*_2_, characterize the growth of drift and diffusion rates and premature sampling noise, respectively. Like Smith and Ratcliff (2009) and Smith et al., (2014), we found that premature sampling can predict fast errors without starting point variability, so we omitted the *s*_*z*_ parameters from the models. The reference model in Table 5 also omits the nondecision time variability parameter *s*_*t*_. Our hypothesis was that the comparatively large *s*_*t*_ estimates found for the standard diffusion model (around 230-260 ms in Table 4 and 200-300 ms in Ratcliff and McKoon (2008)), may be a reflection of the time-varying nature of the process. As we discussed earlier, the standard diffusion model, which represented drift and diffusion rates as random-onset step functions, would characterize data generated by such a process as a distribution of functions with a range of onset times that reflect its growth rate. Although estimates of *s*_*t*_ in the range 200-300 ms are not unusually long compared to those from other tasks (Matzke & Wagenmakers, 2009; Figure A1), it is conceivable that the evidence entering the decision process in these tasks is also time-varying. Two of the most widely studied decision tasks are lexical decision and recognition memory (Ratcliff & Smith, 2004). In these tasks, drift rates are assumed to arise as the result of a matching operation between perception and memory and it is plausible that the information resulting from this operation becomes available gradually rather than in an all-or-none way.

**Table 5:** Time-varying diffusion models

Model	Properties	*m*	Parameters
1	Reference	10	*a*(*s*), *a*(*a*), *ν*(*h*), *ν*(*e*), *π*_*z*_, *η*, *T*_er_, *σ*_2_, *β*, *n*
2	Nonzero *s*_*t*_	11	*a*(*s*), *a*(*a*), *ν*(*h*), *ν*(*e*), *π*_*z*_, *η*, *T*_er_, *s*_*t*_ *σ*_2_, *β*, *n*
3	*a* × *T*_er_	11	*a*(*s*), *a*(*a*), *ν*(*h*), *ν*(*e*), *π*_*z*_, *η*, *T*_er_(*s*), *T*_er_(*a*), *σ*_2_, *β*, *n*
4	*a* × *ν*	12	*a*(*s*), *a*(*a*), *ν*(*hs*), *ν*(*ha*), *ν*(*es*), *ν*(*ea*), *π*_*z*_, *η*, *T*_er_, *σ*_2_, *β*, *n*

*m* = number of free parameters

Even in perceptual tasks, in which stimulus representations can be formed in under 100 ms, the processes that extract the information used to make decisions may be much slower than this. An example is the brightness discrimination task (Ratcliff, 2002; Ratcliff et al., 2003), in which decisions are made about the relative proportions of black and white pixels in briefly flashed, backwardly masked, random pixel arrays. Asymptotic accuracy in this task is attained at exposure durations of around 100 ms (Ratcliff, 2002), consistent with perceptual processing in the Bloch’s law regime, but the estimates of *s*_*t*_ in the diffusion model may range from 110 ms to 170 ms (Matzke & Wagenmakers, 2009). These estimates might seem too long to be attributable to the time course of drift rate formation, but only if drift rate formation is rate-limited by perceptual rather than postperceptual processing. Drift rate in this task presumably arises from a comparison of the encoded perceptual representation with the memory representation of the stimulus attributes that map to the response alternatives. It is unlikely that this comparison can be performed instantaneously, and it seems plausible that it might take several hundred milliseconds to complete.

#### Reference model

Table 6 summarizes the fits of the time-varying models. The first two models in the table are the reference model, which has only a single source of across-trial variability, in drift rate, *η*, and a generalization of the model that includes nondecision time variability, *s*_*t*_. Two things are striking about these fits. First, the time-varying models fit appreciably better than the standard models. For the reference models, the average *G*^2^ of the time-varying model is around to 70% better than that of the standard model. Second, the good fit of the time-varying model was obtained without across-trial variability in nondecision time. Table 7 summarizes the average estimated parameter estimates for the time-varying model, and shows that the average *s*_*t*_ was 24 ms, as compared to 247 ms for the standard model in Table 4. The inclusion of *s*_*t*_ improved the model fit only for a minority of participants: six by the AIC but only one by the BIC. Because the likelihoods in our AIC and BIC statistics have not been adjusted for overdispersion, we regard the more conservative BIC as a more reliable indicator of the performance of the models.

**Table 6:** Fits of time-varying diffusion models

Model	Properties	*G*^2^	*df*	AIC	BIC	#AIC	#BIC
1	Reference	241.94	122	261.94	321.32	—	—
2	Nonzero *s*_*t*_	240.55	121	262.55	327.88	6	1
3	*a* × *T*_er_	236.59	121	25 8.59	323.92	12	6
4	*a* × *ν*	227.85	120	251.85	323.12	16	6

Degrees of freedom not adjusted for individual missing data

**Table 7:** Parameters of time-varying diffusion models

Model	Properties	*a*(*s*)	*a*(*a*)	*ν*(*hs*)	*ν*(*ha*)	*ν*(*es*)	*ν*(*ea*)	*π*_*z*_
1	Reference	0.121	0.195	0.266	—	0.446	—	0.055
2	Nonzero *s*_*t*_	0.122	0.195	0.262	—	0.434	—	0.056
3	*a* × *T*_er_	0.122	0.196	0.268	—	0.450	—	0.055
4	*a* × *ν*	0.119	0.193	0.234	0.256	0.393	0.434	0.056
		*η*	*T*_er_(*s*)	*T*_er_(*a*)	*s*_*t*_	*σ*_2_	*β*	*n*
1	Reference	0.178	0.184	—	—	0.068	24.83	5.08
2	Nonzero *s*_*t*_	0.174	0.173	—	0.024	0.068	28.25	5.75
3	*a* × *T*_er_	0.181	0.183	0.180	—	0.068	25.48	5.25
4	*a* × *ν*	0.162	0.181	—	—	0.066	29.46	5.86

For models with two *μ* parameters *ν*(*ha*) = *ν*(*hs*), *ν*(*ea*) = *ν*(*es*)

**Fig. 6:**
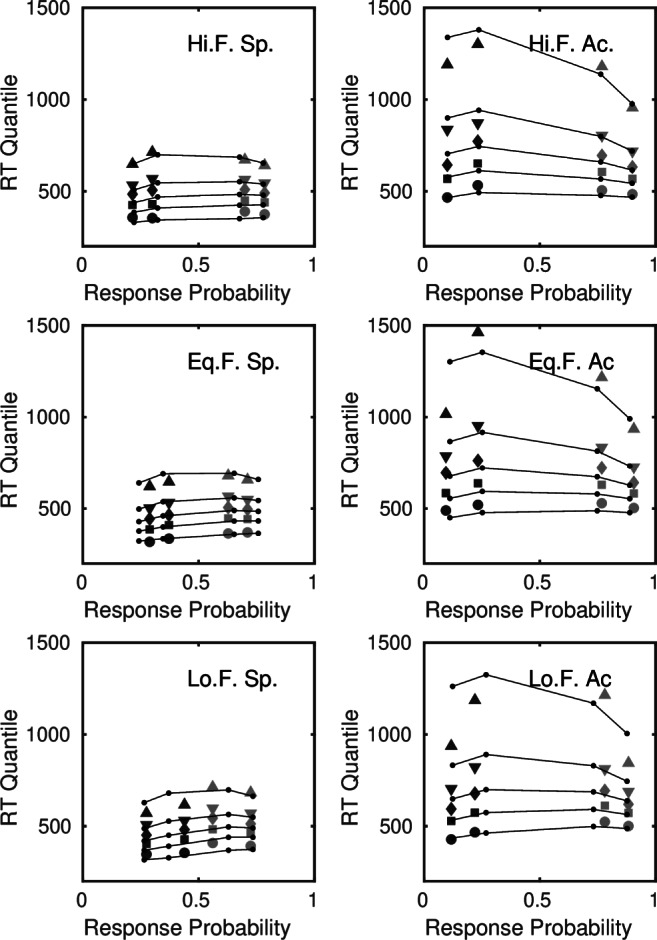
Quantile probability plot of the fit of the time-varying diffusion reference model to participants with complete error data. The columns are speed (Sp.) and accuracy (Ac.) conditions. The *light plotting symbols* are distribution quantiles for correct responses and the *dark symbols* are quantiles for errors

Figure 6 shows a quantile-probability plot of the fit of the reference model to the data of the five participants who had complete error data. Like the standard model, the time-varying model misfits some of the accuracy data, particularly the choice probabilities for low discriminability, low frequency stimuli under speed stress conditions. Where the model performs better than the standard model is in its ability to capture the fast errors in the data, particularly the shift in the .1 quantiles of the error distributions. The model predicts fast errors with no variability in starting point, via premature sampling noise, *σ*_2_. The estimated value of 0.068 in Table 7 implies that, asymptotically, premature sampling contributed around 30% of the noise in the evidence accumulation process.

#### Selective influence violation models

The other models in Tables 5, 6 and 7 are selective influence violation models. Because the nondecision variability effects were so small for the time-varying model, we did not consider the *a* × *s*_*t*_ model, which allowed nondecision time variability to depend on speed versus accuracy instructions. Model 3, *a* × *T*_er_, allowed mean nondecision times to vary with instructions. Table 6 shows that the *a* × *T*_er_ model was preferred to the reference model for 12 of the participants according to the AIC, but only for six of them by the BIC. This compares with the corresponding figures of 12 and 11 for the standard diffusion model in Table 3. If we accept that the picture provided by the BIC is likely to be the more reliable one, then this implies that the selective influence violations involving nondecision times were less prevalent for the time-varying model. The averaged estimated *T*_er_ values for the standard model in Table 4 are *T*_er_(*s*) = 254 ms and *T*_er_(*a*) = 270 ms. The corresponding estimates for the time-varying model in Table 7 are *T*_er_(*s*) = 183 ms and *T*_er_(*a*) = 180 ms. At the group level, the first of these differences is highly significant by a (classical) matched-pairs *t*-test, *D* = 16 ms, *t*(19) = 4.33, *p* < .0005, whereas the second of them is not, *D* = − 3 ms, *t*(19) = − 1.73, *p* > 0.05. The ordering of the *T*_er_ estimates for the time-varying model is the opposite of the one found for the standard model here and elsewhere, and when we refit the *a* × *T*_er_ model with the ordinal constraint *T*_er_(*a*) ≥ *T*_er_(*s*), only one of the participants was better fit by the model than by the reference model according to the BIC, although a similar number were better fit by the AIC. Taken together, the model selection statistics and the group tests of the estimated effects show that selective influence violations involving nondecision times, although not completely eliminated in the time-varying model, were smaller and less systematic.

The fourth model, *a* × *ν*, is a selective influence violation model that tested whether mean drift rates were the same under speed and accuracy instructions. For the standard diffusion model, the model *a* × *ν* was preferred for 11 participants according to the AIC and for eight according to the BIC. For the time-varying model, the corresponding numbers were 16 and 6. (Overall, a selective influence violation model was preferred for 18 participants by the AIC and eight by the BIC.) For both models, the estimated mean drift rates were larger under accuracy instructions than under speed instructions. For the standard diffusion model, the drift rates were *ν*(*hs*) = 0.153, *ν*(*ha*) = 0.174, *ν*(*es*) = 0.272, and *ν*(*ea*) = 0.316; for the time-varying model they were *ν*(*hs*) = 0.234, *ν*(*ha*) = 0.256, *ν*(*es*) = 0.393, and *ν*(*ea*) = 0.434. At a group level, all effects for both models were highly significant by a repeated-measures ANOVA. For the standard model, the ANOVA yielded *F*_ease_(1,19) = 199.9, *p* < 1.0 × 10^− 10^; *F*_speed_(1,19) = 20.45, *p* < 0.0005, and *F*_ease×speed_(1,19) = 15.57, *p* < 0.001. For the time-varying model, it yielded *F*_ease_(1,19) = 693.9, *p* < 1.0 × 10^− 15^; *F*_speed_(1,19) = 5.79, *p* < 0.05, and *F*_ease×speed_(1,19) = 6.01, *p* < 0.05. For both models, mean drift rates were higher for easy than for difficult stimuli, as expected; but, contrary to selective influence assumptions, they were also higher under accuracy than speed instructions and, moreover, the difference between easy and difficult stimuli was increased under accuracy instructions.

The model selection statistics, especially the BIC, suggest that selective influence violations involving mean drift rates may arise only for a subset of participants, but when they do occur they are of sufficient magnitude to yield highly significant group-level effects. Our hypothesis was that these kinds of violations might reflect the time-dependent nature of the evidence accumulation process. If evidence accumulation is described by Eq. 4, in which the signal-to-noise ratio, $\mu \theta (t)/\sqrt {{\sigma _{1}^{2}}\theta (t) + {\sigma _{2}^{2}}}$, increases over the course of trial, then the effective signal-to-noise ratio will be lower under speed instructions when decision boundaries are narrower, because decisions will be more dependent on evidence sampled early in a trial. Our hypothesis was that, if these effects are characterized using the standard diffusion model, in which the signal-to-noise ratio is constant, then the estimated drift rates in the model would be lower under speed instructions. Although this dependence of drift rates on instructions is a mathematical consequence of Eq. 8, the magnitude of the effect in fitting the data was not sufficient to eliminate the selective influence violations represented by model *a* × *ν*.

#### Sampling precision models

Our finding that mean drift rates in the time-varying model were higher under accuracy than under speed instructions, even after the changes in stimulus signal-to-noise ratios during the course of a trial were taken into account, led us to look for ways to modify the model that might explain these effects in a principled way. This led to a class of models we call *sampling precision* models. The idea behind them is that the imperative to go fast may cause people to form less precise cognitive representations of the stimuli about which they are making decisions. This kind of variation may be attentional in origin: Attempting to go fast makes people attend less to the fine detail of stimuli. One way to formalize this idea in a time-varying framework is to assume that the stimulus-independent diffusion noise, *σ*_2_, varies with speed-versus-accuracy instructions. The most parsimonious way to formalize this idea is to assume that the total diffusion rate remains constant across instructions, but that the relative proportions of stimulus-dependent and stimulus-independent diffusion change. This constraint can be realized by imposing appropriate restrictions on the diffusion terms in the evidence accumulation equation, Eq. 4,

10 $$ {\sigma_{1}^{2}}(s) + {\sigma_{2}^{2}}(s) = {\sigma_{1}^{2}}(a) + {\sigma_{2}^{2}}(a); \sigma_{1}(a) = 0.1,  $$

with *σ*_2_(*s*) and *σ*_2_(*a*) free parameters to be estimated from the data. The condition *σ*_1_(*a*) = 0.1 sets the overall scale of the model, which is required to make it identifiable, like the other models we have considered. When the diffusion rates are restricted in this way, the model has one more free parameter than the reference model in Tables 5, 6 and 7.

Table 8 shows the fit of the sampling precision model *a* × *σ*_2_ and Table 9 shows the estimated parameters. The tables reproduce the fit statistics and parameters for the reference model for comparison purposes. The inclusion of sampling precision effects led to a substantial improvement in model fit: Nineteen participants were better fit by a model with sampling precision effects according to the AIC and 14 were better fit according to the BIC. The parameter estimates in Table 9 showed that, on average, *σ*_2_(*s*) was larger than *σ*_2_(*a*). Averaged over all participants, the stimulus-independent diffusion terms for the speed and accuracy conditions were *σ*_2_(*s*) = 0.064 and *σ*_2_(*a*) = 0.062, and for the 14 participants who were better fit by the sampling precision model according to the BIC, the corresponding estimates were *σ*_2_(*s*) = 0.069 and *σ*_2_(*a*) = 0.064. Although the *σ*_2_ estimates for both the full sample and the subsample appear fairly similar numerically, small changes in diffusion rate can have substantial effects on predicted RT distributions, including on the location of the .1 quantile (Donkin et al., 2009; Smith et al., 2014).

**Table 8:** Fits of sampling precision models

Model	Properties	*G*^2^	*df*	AIC	BIC	#AIC	#BIC
1	Reference	241.94	122	261.94	321.32	—	—
2	*a* × *σ*_2_	225.67	121	247.67	313.00	19	14
3	*a* × *σ*_2_ + *a* × *ν*	214.34	119	240.34	317.59	11	6

**Table 9:** Parameters of sampling precision models

Model	Properties	*a*(*s*)	*a*(*a*)	*ν*(*hs*)	*ν*(*ha*)	*ν*(*es*)	*ν*(*ea*)	*π*_*z*_
1	Reference	0.121	0.195	0.266	—	0.446	—	0.055
2	*a* × *σ*_2_	0.120	0.191	0.252	—	0.420	—	0.054
3	*a* × *σ*_2_ + *a* × *ν*	0.120	0.193	0.229	0.253	0.382	0.426	0.054
		*η*	*T*_er_	*σ*_2_(*s*)	*σ*_2_(*a*)	*β*	*n*	
1	Reference	0.178	0.184	0.068	—	24.83	5.08	
2	*a* × *σ*_2_	0.163	0.162	0.064	0.062	28.81	6.42	
3	*a* × *σ*_2_ + *a* × *ν*	0.158	0.162	0.064	0.062	30.58	6.70	

Our main interest in sampling precision models was in whether allowing *σ*_2_ to vary with instructions would eliminate their effect on mean drift rates. To this end, we also looked at the model *a* × *σ*_2_ + *a* × *ν*, which allowed both diffusion noise and mean drift rate to vary with instructions. Unlike the corresponding entries in Tables 4 and 6, the figures in the columns #AIC and #BIC in Table 8 are the numbers of participants who were better characterized by model *a* × *σ*_2_ + *a* × *ν* than by model *a* × *σ*_2_, that is, by a model in which instructions affected both mean drift rate and diffusion noise rather than noise alone. By the AIC and BIC, there were 11 and 6 such participants, respectively, as compared to the 16 and 6 for model *a* × *ν* in Table 6. Inclusion of sampling precision in the model therefore appears to reduce selective influence violations involving mean drift rates, but does not eliminate them.

At a group level, the effects involving differences in mean drift rates among conditions were substantial. In a repeated measures ANOVA on the mean drift rates for model *a* × *σ*_2_ + *a* × *ν*, both the main effects of ease and speed and their interaction were significant: *F*_ease_(1,19) = 612.3, *p* < 1.0 × 10^− 10^; *F*_speed_(1,19) = 7.72, *p* < 0.05, and *F*_ease×speed_(1,19) = 6.44, *p* < 0.05. The effect of speed on mean drift rates remained significant when the analysis was restricted to the subsample of 14 participants for whom the BIC-preferred model did not include the *a* × *ν* interaction term: *F*_ease_(1,13) = 347.6, *p* < 1.0 × 10^− 10^; *F*_speed_(1,13) = 4.77, *p* < 0.05, and *F*_ease×speed_(1,13) = 4.27, *p* > 0.05. The effect size for speed is almost the same for the subsample, ${\eta _{p}^{2}} = 0.268$, as for the whole sample, ${\eta _{p}^{2}} = 0.289$. The group results therefore suggest that, in addition to affecting diffusion noise, speed instructions also have a direct effect on mean drift rates.

#### Evidence growth functions

**Fig. 7:**
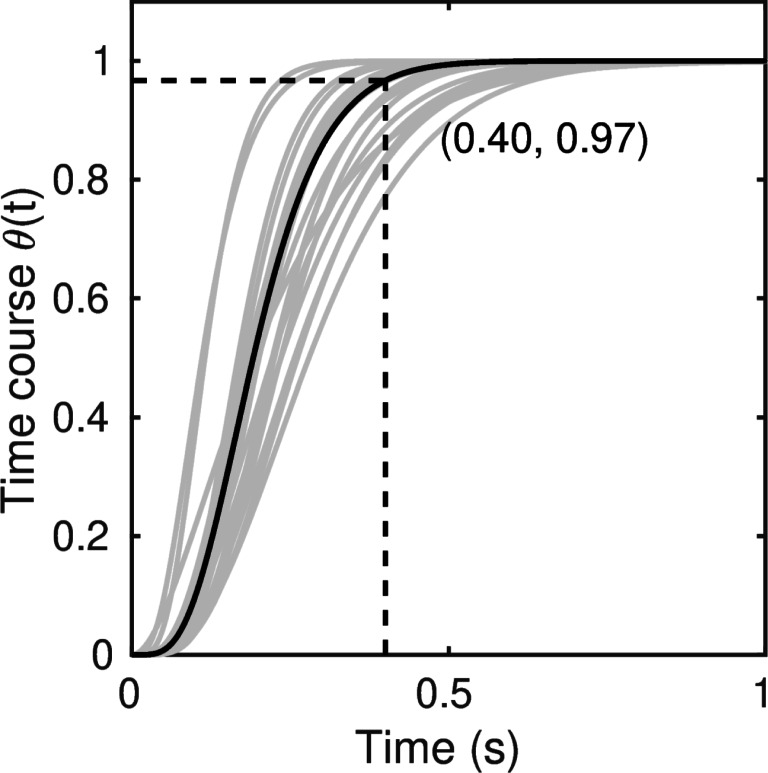
Evidence growth function, *𝜃*(*t*), for drift and diffusion rates in the time-varying reference model. The *light lines* are the estimated functions for the individual participants and the *dark line* is the function for the parameters $\bar {\beta }$, and $\bar {n}$, averaged across participants

The fits of the time-varying models yield estimates of the function *𝜃*(*t*) in Eq. 9, which, when used in Eq. 4, describe the growth in drift and diffusion rates over time. Figure 7 shows estimates of *𝜃*(*t*) for the individual participants, together with a group function based on averages of the parameters of the individual participants, $\bar {\beta } = {\sum }_{j}\beta _{j}/20$ and $\bar {n} = {\sum }_{j} n_{j}/20$. The estimates of evidence growth are in remarkably good agreement with the temporal integration times for the RDM task found by Watamaniuk and Sekuler (1992) and reproduced in Fig. 2. Notably, Fig. 7 shows that *𝜃*(*t*) attains its maximum at around 400 ms or a little later. At 400 ms, the function has attained 97% of its asymptotic value. Watamaniuk and Sekuler showed that discrimination accuracy improved with increasing stimulus duration up to around 400-450 ms. The functions in Fig. 7 show that, for the response-terminated stimuli used in the Dutilh et al., (2019) study, the signal-to-noise ratio of the evidence entering the decision process, as expressed by the ratio of the drift and diffusion rates, progressively increases during the first 400 ms or so, but is constant thereafter. The fact that two quite different experimental paradigms using the RDM task should have yielded such consistent estimates of the underlying temporal integration processes is striking and is evidence of the convergent validity of the time-varying diffusion model.

#### Correlations among parameters

Like the standard model, the relationships among the parameters of the time-varying model are of theoretical interest. Table 10 shows the correlations among the main parameters of the reference model. To summarize the growth rate function *𝜃*(*t*), we used the ratio of the shape and rate parameters, *𝜃*_*ν*_ = *n*/*β*, in Eq. 9. When Eq. 9 is interpreted as a probability distribution, *𝜃*_*ν*_ is the mean of the distribution. When *n* is an integer, the mean is equal to the number of exponential stages in cascade, multiplied by the stage mean 1/*β*. When the incomplete gamma is interpreted as the output of a linear system, as here, the ratio can be interpreted as a system rate constant, which characterizes how rapidly the output changes over time.

**Table 10:** Time-varying model parameter correlation matrix

	*a*(*s*)		*a*(*a*)		*ν*(*h*)		*ν*(*e*)		*𝜃*_*ν*_	*T*_er_	
*a*(*s*)			0.071		0.538	*	0.616	**	-0.198	-0.223	
*a*(*a*)					-0.074		-0.018		0.346	-0.063	
*ν*(*h*)							0.946	***	-0.076	-0.310	
*ν*(*e*)									-0.143	-0.215	
*𝜃*_*ν*_										-0.473	*
*T*_er_											

∗ = *p* < .05; ∗∗ = *p* < .01; ∗∗∗ = *p* < .001

The most important result in Table 10 is that the growth of drift and diffusion rates, *𝜃*_*ν*_, is not significantly correlated with either boundary separation or mean drift rate. This is consistent with the picture from the Watamaniuk and Sekuler (1992) study, in which the critical durations were the same for high and low coherence stimuli. Complementing their findings, our results show that asymptotic stimulus discriminability, which depends on the drift rate parameters *ν*(*s*) and *ν*(*a*), and the amount of evidence used to make a decision, *a*(*s*) and *a*(*a*), are unrelated to the rate at which stimulus information becomes available. Unsurprisingly, *T*_er_ and *𝜃*_*ν*_ were significantly negatively correlated: The value of *T*_er_ is the point at which the drift and diffusion terms change from zero to small, nonzero values, and we would expect this point to be difficult to identify empirically and to lead to trade-offs in estimation. The negative correlation is a reflection of this difficulty.

The estimates of mean drift rates in the easy and difficult condition were highly correlated with one another, indicating that stimulus discriminability is a significant individual differences variable, as we would expect in a near-threshold perceptual task. Surprisingly, boundary separations in the speed and accuracy settings were uncorrelated, suggesting that there is no corresponding individual differences variable of response caution governing decision strategies in the two instruction conditions. The mean drift rates, *ν*(*h*) and *ν*(*e*), were significantly correlated with boundary separation in the speed condition only, but were uncorrelated with boundary separation in the accuracy condition. These correlations appear to be another expression of the *a* × *ν* selective influence violation, in which estimates of drift rate are higher in participants with wider boundaries, at least under speed-stress conditions. There were similar correlations for the standard diffusion model: For the reference model in Tables 2 and 4, the correlations of *ν*(*h*) and *ν*(*e*) with *a*(*s*) were *r* = .634, *p* < 0.001 and *r* = 0.698, *p* < 0.001, respectively, but the correlations in the accuracy condition were nonsignificant.

Table 11 provides a summary of all of the models we compared, rank-ordered by average BIC. The models fall into three clear, nonoverlapping groups. On average, the best models were the sampling-precision models; the next best were the time-varying models with no sampling precision effects, and the poorest were the standard diffusion models. The best model overall was the time-varying sampling precision model, *a* × *σ*_2_, in which premature-sampling noise varied with experimental instructions.

**Table 11:** Rank-ordering of models by BIC

Rank	Model	*G*^2^	*df*	BIC
1	Sampling Precision *a* × *σ*_2_	225.67	121	313.00
2	Sampling Precision *a* × *σ*_2_ + *a* × *ν*	214.34	119	317.59
3	Time-Varying Reference	241.94	122	321.32
4	Time-Varying *a* × *ν*	227.85	120	323.12
5	Time-Varying *a* × *T*_er_	236.59	121	323.92
6	Time-Varying Nonzero *s*_*t*_	240.55	121	327.88
7	Standard Diffusion *a* × *s*_*t*_	385.47	121	472.80
8	Standard Diffusion *a* × *T*_er_	392.84	121	480.17
9	Standard Diffusion *a* × *ν*	390.16	120	485.43
10	Standard Diffusion Reference	410.91	122	491.72
11	Standard Diffusion Drift Criterion	405.45	121	492.78

## Discussion

Our study was motivated by the Dutilh et al., (2019) finding of a pervasive failure of selective influence on nondecision times in the standard diffusion model. They argued that these failures may be real effects and that nondecision times may be affected by instructions to be either fast or accurate. Our interpretation of these failures was that they may be an artifact of fitting data from a time-varying process with a time-homogeneous model. We based our argument on the TvD curves for the RDM task, which show atypically long critical durations. One interpretation of the critical duration is that it characterizes the time during which the perceptual system forms a global representation of motion from the local motion vectors of the individual dots. If this interpretation is correct—and to us it is the most plausible one—then it suggests that the formation of drift rates may take several hundred milliseconds. If so, then decisions in the RDM task may be better characterized by a time-inhomogeneous model, in which drift and diffusion rates progressively increase over time, than by a time-homogeneous one in which they are represented as random step functions. The aim of our study was to investigate a model of this kind.

The researchers in the Dutilh et al., (2019) study were set the challenging task of inferring experimental manipulations from blinded, two-condition, experimental designs, and we wondered whether there was enough structure in such minimal designs to allow model parameters to be recovered reliably. We therefore fit the data from the full experimental design using the standard diffusion model, making judicious use of selective influence assumptions to constrain the space of models to something manageable. Like Dutilh’s researchers, we found violations of selective influence involving both mean nondecision time, *T*_er_, and nondecision time variability, *s*_*t*_, as well as mean drift rates, *ν*. We concluded that the violations of selective influence found by Dutilh et al. were not artifacts of inference from minimal designs, but were instead a property of the RDM task itself.

In the second part of our study, we compared the standard diffusion model to a time-varying model based on the integrated system model of Smith and Ratcliff (2009), in which the evidence entering the decision process depends on the output of time-varying visual filters. First, the fits of the time-varying model were appreciably better than those of the standard diffusion model. Second, we were able to fit the time-varying model using only one source of across-trial variability in the model rather than three.

The better fit of the time-varying model does not appear to be simply a matter of relative model flexibility, but, rather, seems to be a reflection of how evidence enters the decision process, which the model captures better than does the standard model. If the onset of evidence accumulation were abrupt, as the standard model assumes, then this could be represented in the time-varying model by choosing the parameters *β* and *n* so that *𝜃*(*t*) approximates a step function.^5^ However, this representation would require two more parameters than the standard model to represent the same properties. In addition, the effects of the two diffusion terms, *σ*_1_ and *σ*_2_, would then be indistinguishable, so the model could not predict fast errors. Under these conditions, the three unique parameters of the time-varying model become redundant, so we would expect its performance as assessed by the AIC or BIC to be worse than that of the standard model. That this was not the case implies these parameters of the time-varying model are capturing features of the data that the standard model does not.

Theoretical questions are rarely resolved on the basis of goodness-of-fit alone, and other researchers, notably Ratcliff and McKoon (2008), have obtained excellent fits of the standard diffusion model to data from the RDM task. Nevertheless, the quality of the fits we obtained for the time-varying model to the Dutilh et al., (2019) data is encouraging. The only source of across-trial variability in our model was in drift rate, *η*, which accounted for the differences in the upper quantiles of the RT distributions for correct responses and errors under accuracy instructions. The fast errors under speed instructions and the shift in the .1 error distribution quantiles under both forms of instruction were accounted for by stimulus-independent diffusion noise, of the kind that Laming (1968) attributed to premature sampling. Premature sampling in the RDM task, as in other dynamic noise tasks, is a plausible psychological consequence of a process in which discriminative information only becomes available progressively, some time after stimulus onset.

Another way to predict fast errors was recently proposed by Voss et al., (2019), who assumed that, rather than evidence being accumulated by a diffusion process, it is accumulated by a Lévy process. A Lévy process is a stochastic process composed of a superposition of a continuous, diffusion-like process and a jump process, like a Poisson process, in which the jumps are of varying magnitudes (Bertoin, 1996). The presence of jumps in the process increases the likelihood of random boundary crossings early in evidence accumulation and allows the model to predict fast errors. However, Voss et al. provided no strong arguments for why evidence accumulation should be represented cognitively by the more complex Lévy process, apart from fact that it can predict fast errors. In contrast, the model proposed here requires no change in the standard assumption that evidence accumulation is represented by a diffusion process. Also, unlike the model of Voss et al., whose predictions have no explicit mathematical form and must be obtained by Monte Carlo simulation, the predictions for the time-varying diffusion model are mathematically explicit and computationally tractable.

Unlike the standard diffusion model, we were able to characterize the RT distributions in the Dutilh et al., (2019) data using a time-varying model without variability in nondecision time, *s*_*t*_. For a number of well-studied decision tasks, the fit of the standard model is appreciably improved if the nondecision time, *T*_er_, is assumed to be random rather than fixed (Matzke and Wagenmakers, 2009). The leading edges of the RT distributions predicted by the standard model are often sharper than those in empirical distributions and fits are improved by treating *T*_er_ as a random variable rather than as fixed. Estimates of *T*_er_ variability in the RDM task are usually fairly large, but we showed that, for most participants, the *s*_*t*_ component of variability could be omitted from the model without worsening the fit. Although the time-varying model is more complex than the standard model in its assumptions about drift and diffusion rates, this complexity is offset by gains in parsimony elsewhere—specifically, the fact that we were able to fit the model using only a single source of across-trial trial variability.

Our working hypothesis was that the violations of selective influence in the standard model found by Dutilh et al., (2019) may be artifacts of the time-varying nature of the decision process. If so, then we expected that these effects would be eliminated by using a model that takes the time course of stimulus processing into account. This hypothesis was partially, but not completely, supported. For the standard model, we found a substantial number of selective influence violations involving both mean nondecision time, *T*_er_, and nondecision time variability, *s*_*t*_. For the time-varying model, we found the number of violations of selective influence involving *T*_er_ was reduced—at least according to the BIC—and they were greatly reduced in magnitude, and we were able to fit the model with no *s*_*t*_ variability. However, the violations involving mean drift rate, *ν*, were more persistent. According to the BIC, these violations were present for less than half the participants, but they were large enough to produce highly significant group-level differences. Our hypothesis was these violations may be due to the time-varying nature of the decision process interacting with differences in the total amount of information sampled under speed and accuracy instructions. Contrary to our hypothesis, however, these violations of selective influence were also found for the time-varying model.

To explain them, we proposed an elaboration of the time-varying model that assumed that the instruction to go fast leads to a loss in sampling precision in the perceptual encoding of stimuli, which we suggested may be attentional in origin. Reduced sampling precision under speed instructions is represented in the model by a change in the relative proportions of stimulus-dependent and stimulus-independent diffusion noise. The consequence of a loss of sampling precision is to make participants more prone to premature sampling under speed instructions. The inclusion of instruction-dependent sampling noise, *a* × *σ*_2_, in the model improved the fit for the majority of participants, but there was a minority for whom it was further improved by inclusion of the *a* × *ν* interaction. These effects were found only for some participants, but, as they also have been reported by previous authors, we think they are likely to be real ones. For those participants for whom model *a* × *σ*_2_ + *a* × *ν* was the preferred model, the fits imply that instructions affected both the mean and the noisiness of the stimulus information entering the decision process.

Apart from the overall quality of the fit, one of the most persuasive pieces of the evidence for the time-varying model is the estimated evidence growth function, *𝜃*(*t*), in Fig. 7. The figure shows that the evidence entering the decision process grows during the first 400 ms after stimulus onset and then reaches an asymptote. This estimate agrees nicely with the temporal integration time for the RDM task reported by Watamaniuk and Sekuler (1992) and reproduced in Fig. 2. Although there have been other estimates of the critical duration in the RDM task, and some authors have found no evidence for a critical duration, several studies have corroborated Watamaniuk and Sekuler’s estimate and, qualitatively, their data appear particularly regular and compelling. The 400 ms critical duration also agrees with the piecewise LBA fits of Holmes et al., (2016), who found the change in drift rates estimated from the model lagged the change in the stimulus by around 400 ms.

The finding that the reduction in coherence thresholds in the RDM follows a square-root law (Watamaniuk, 1993) suggests that the computation of drift rate may involve some form of averaging or weighted averaging of local motion vectors up to the critical duration. Evidence for such an averaging process was recently provided by Ratcliff and Smith (2020) who studied performance in the RDM task at a range of different stimulus exposure durations. They reported the counterintuitive finding that accuracy increased with exposure duration, as expected, but that RTs, instead of becoming shorter, became longer. This pattern of RT and accuracy could be captured in the standard diffusion model by assuming that mean drift rates were constant, or relatively constant, after the first 100 ms of exposure, but that drift rate standard deviation, *η*, progressively decreased throughout the first 400 ms. A decrease in *η* would be predicted if drift rates depend on the average of noisy motion vectors within a fixed temporal window, because *η* would then be proportional to the standard error of the mean. These RT and accuracy properties can also be captured by a version of the dynamic noise model of Smith et al., (2014), but at the cost of making more complex representational assumptions than the ones we have made here. Our goal here was not to provide a process model of drift rates in the RDM task, but to compare constant and time-varying drift rate models using the fewest assumptions.

A larger aim of our study was to serve as a piece of advocacy for time-varying models. Despite the widespread use of visual tasks in the study of evidence accumulation models, the field as a whole has shown little interest in the temporal properties of the evidence entering the decision process. This is despite the existence of an established literature on visual temporal sensitivity that has developed methods for characterizing it in detail (Gorea and Tyler, 1986; Watson, 1986). The consistent message to have come out of this literature is that there are no step functions in vision. This message is at odds with the majority of evidence accumulation decision models that assume time-homogeneous evidence accumulation.

There are two likely reasons for the lack of interest in fine-grained temporal dynamics among decision researchers. One is the notable success of time-homogeneous decision models in accounting for a large body of experimental data, to a degree that has few parallels elsewhere in psychology. The other is an understandable wish not to further complicate what are already complex models. However, failures of selective influence like those reported by Dutilh et al., (2019) call this pragmatic stance into question. There has been an increasing tendency in the field to equate violations of selective influence—or violations of particular authors’ interpretations of what the selective influence assumptions should be (Jones and Dzhafarov, 2014; Sun & Landy, 2016)—with a failure of the model as a whole. This, to us, is the wrong interpretation of such failures. We believe the correct interpretation is to acknowledge that the standard diffusion model (or the standard LBA) is likely to be at best an approximation that will work well when the temporal dynamics of the task are fast, but that will break down when they are slow. We have argued that the psychophysical evidence suggests that the dynamics of the RDM task are slow. To go beyond the simple empirical finding of a violation of selective influence to an understanding of its cause requires us to enlarge the model space. The most productive way to do this, we believe, is to develop submodels of the processes that compute the evidence entering the decision process. In such an enriched framework, models can act as lenses that allow us to ask and answer very focused questions about underlying processes. Our sampling precision model embodies the kind of focused, theory-driven question that can be formulated in this way. Further selective-influence studies that provide more examples of violations of assumptions in an atheoretical way are likely to be unproductive.

There are, potentially, further benefits to thinking about the decision process in the RDM task as time-varying rather than time-homogeneous. An issue that cuts across the issue of selective influence violations considered in this article is the issue of “collapsing decision bounds.” A question that has occasioned lively debate in the recent literature is whether decision boundaries remain constant or decrease (i.e., converge) during the course of a trial. The debate, and the evidence that has been marshalled on both sides of it, takes in neuroscience (Gold & Shadlen, 2003), mathematical optimality theory (Drugowitsch et al., 2012; Malhotra et al., 2018), and computational modeling (Hawkins et al., 2015; Palestro et al., 2018; Voskuilen et al., 2016; Voss et al., 2019). The relevance of this debate to our current study is that many of the studies that have yielded evidence for collapsing decision bounds have used the RDM task (Hawkins et al., 2015; Palestro et al., 2018). Model comparison studies have compared fixed and collapsing-bounds versions of the diffusion model in which the drift and diffusion rates are constant within a trial, but our analysis suggests that these models may be too limited to be truly diagnostic of the underlying processes.

We have carried out simulations of a time-varying diffusion model described by Eq. 2 and the growth rate function of Eq. 8 and fit the simulated data with fixed-bound and collapsing-bound decision models. We used the integral equations of Voskuilen et al. (2016, Appendix B) to generate predictions and hyperbolic decision boundaries similar to those typically used to characterize neural data (Churchland et al., 2008; Hanks et al., 2011; Voskuilen et al., 2016). We found that a process with time-varying drift rates and fixed bounds was better fit in all cases by a model with collapsing bounds if there was no across-trial variability in the model. However, if there was across-trial variability in nondecision time with *s*_*t*_ of around 200 ms, then a fixed-bound model tended to be preferred. In either instance, the resulting fits are a reflection of the time-varying nature of drift and diffusion rates. When there is no *s*_*t*_ term in the model, time-varying drift and diffusion rates are misidentified as collapsing boundary effects, but if *s*_*t*_ is included, then these changes can be compensated for, at least in part, by allowing the onset time of the decision process to be random. We think that the selective influence violations involving *s*_*t*_ in the standard diffusion model we found here are a product of the same kind of compensation process.

The issue of collapsing versus fixed decision bounds raises several theoretical issues that are beyond the scope of this article, and which we take up elsewhere, such as whether, and under what circumstances, a model with collapsing bounds is equivalent to one with a time-varying “urgency signal” (Churchland et al., 2008), possibly acting in concert with novelty-based stimulus encoding (Cisek et al., 2009). Our point here is simply that we believe the collapsing-bounds debate, like the selective influence debate, has been less illuminating than it might have been otherwise because it has restricted itself to a limited set of theoretical alternatives.

## Conclusions

In this article we re-examined the pervasive evidence of selective influence violations found in the Dutilh et al., (2019) blinded model validation study. We hypothesized that the violations may be a reflection of the psychophysical properties of the RDM task itself, which has slow temporal dynamics, and refitted the full set of data using a model in which drift and diffusion rates increased progressively over time. The time-varying model yielded a better fit to the data than did the standard diffusion model, and was able to account for the data using only a single source of across-trial variability rather than three. Estimates of the time course of the evidence entering the decision process yielded an integration time of around 400 ms, in good agreement with estimates of the critical duration in the RDM task in the visual psychophysics literature. Although violations of selective influence in the time-varying model were not eliminated, they were reduced relative to the standard model. Our study suggests that the standard diffusion model, which assumes abrupt-onset drift and diffusion rates, may provide a good description of performance in tasks in which the time course of stimulus processing is fast, but may have difficulty with tasks like the RDM task, in which the time course of stimulus processing is slow. These difficulties may manifest themselves as violations of selective influence. Instead of further atheoretical selective influence studies, we argue that the field would most benefit by considering an enlarged model space, in which the time course of the evidence entering the decision process is characterized theoretically and modeled in an explicit way.

## Appendix A: Integral-equation representations of first-passage time densities for time-varying diffusion models

In this appendix we outline the derivation of the kernel of the integral equation, Ψ(*a*_*i*_,*t*|*a*_*j*_,*τ*), in Eq. 7 and give the discretized forms of Eqs. 5 and 6 that we used to generate predictions for our time-varying model. A comprehensive tutorial introduction to the integral equation method providing full mathematical details may be found in Smith (2000).

The most general diffusion process, *X*_*t*_, is governed by a stochastic differential equation of the form

A1 $$ dX_{t} = \mu(x, t)  dt + \sigma(x, t)  dW_{t},  $$

in which the drift rate and diffusion coefficient depend both on time, *t*, and on the position of the process in the evidence space, *x*. Buonocore et al., (1990) showed that for a large class of diffusions the kernel of the integral equations has a particular form, given in Eq. A7 below, which depends on the existence of a coordinate mapping that transforms the process *X*_*t*_ into a standard Wiener, or Brownian motion, process, with zero drift rate and unit variance. The state and time coordinates, *x*^∗^ and *t*^∗^, of the transformed process are related to the coordinates of the original process, *x* and *t*, by a pair of functions

A2 $$ \begin{array}{@{}rcl@{}} x^{*} & = & \bar{{\Psi}}(x, t) \end{array} $$

A3 $$ \begin{array}{@{}rcl@{}} t^{*} & = & {\Phi}(t). \end{array} $$

The new state coordinate is a function jointly of the old state and time coordinates, while the new time coordinate is a function of the old time coordinate alone. (Note carefully the overbar notation in Eq. A2, which distinguishes the state mapping variable function from the kernel function itself.)

The existence of the coordinate transformation in Eqs. A2 and A3 depends on the existence of a pair of functions *c*_1_(*t*) and *c*_2_(*t*) of time only, which relate the drift and diffusion coefficients of the process described by Eq. A1 in a prescribed way. For the general equation A1, the expression relating the drift and diffusion coefficients is somewhat complicated (Ricciardi and Sato, 1983, Smith, 2000, Equation 48), but for the special case in which the drift rate is *μ*(*x*,*t*) and the diffusion coefficient is *σ*^2^(*t*), that is, in which the drift rate may depend on both state and time but the diffusion coefficient depends only on time, the relation is simpler (Smith et al.,, 2014; Appendix B). Specifically, it is

A4 $$ \mu(x, t) = \frac{\sigma(t)}{2} c_{1}(t) + \frac{x}{2}\left[c_{2}(t) + \frac{\sigma^{2\prime}(t)} {\sigma^{2}(t)}\right].  $$

In this equation, $\sigma ^{2\prime }(t)$ is the derivative of the diffusion coefficient with respect to time. If functions *c*_1_(*t*) and *c*_2_(*t*) can be found that satisfy this equation, then the functions transforming the process *X*_*t*_ into a zero-drift, unit variance, Wiener process have the form

A5 $$ \begin{array}{@{}rcl@{}} x^{*} & = & \bar{{\Psi}}(x,t) = \exp\left[-\frac{1}{2}{\int}^{t} c_{2}(s) ds\right] {\int}^{x} \frac{dy}{\sigma(t)} \\ & & -\frac{1}{2}{\int}^{t} c_{1}(s) \exp\left[-\frac{1}{2}{\int}^{s} c_{2}(z) dz\right] ds \end{array} $$

A6 $$ \begin{array}{@{}rcl@{}} t^{*} & = & {\Phi}(t) = {\int}^{t}\exp\left[-{\int}^{s} c_{2}(z)  dz\right] ds. \end{array} $$

For a diffusion process with fixed boundaries, *a*_*i*_, *i* = 1, 2, the kernel of the integral equation Ψ(*a*_*i*_,*t*|*a*_*j*_,*τ*) in Eq. 7 can be written in terms of the coordinate transformation functions (Gutiérrez Jáimez et al.,, 1995; Smith, 2000, Equation 56) as

A7 $$ \begin{array}{@{}rcl@{}} {\Psi}(a_{i}, t  |  a_{j}, \tau) \!& = & \frac{f(a_{i}, t  |  a_{j}, \tau)}{2} \\ &&\times\! \left\{\!\frac{\bar{{\Psi}}_{t}^{\prime}(a_{i}, t)}{\bar{{\Psi}}_{x}^{\prime}(a_{i}, t)} - \frac{\left[\bar{{\Psi}}(a_{i}, t) - \bar{{\Psi}}(a_{j}, \tau)\right]} {{\Phi}(t)\! -\! {\Phi}(\tau)} \frac{{\Phi}^{\prime}(t)}{\bar{{\Psi}}_{x}^{\prime}(a_{i},t)}\!\right\}.\\ \end{array} $$

In this equation, $\bar {{\Psi }}_{x}^{\prime }(a_{i}, t)$ and $\bar {{\Psi }}_{t}^{\prime }(a_{i}, t)$ are the partial derivatives of $\bar {{\Psi }}(\cdot )$ with respect to state and time, respectively, and ${\Phi }^{\prime }(t)$ is the derivative of Φ(⋅) with respect to time. The function *f*(*a*_*i*_,*t*|*a*_*j*_,*τ*) is the transition density of the process *X*_*t*_, unconstrained by boundaries, expressed in terms of the functions that transform the process from the old to the new coordinates (Smith, 2000, Equation 51),

A8 $$ \begin{array}{@{}rcl@{}} f(a_{i}, t  |  a_{j}, \tau) &=& \frac{1}{\sqrt{2\pi[{\Phi}(t) - {\Phi}(\tau)]}} \\ &&\exp\left\{ -\frac{[\bar{{\Psi}}(a_{i},t) - \bar{{\Psi}}(a_{j}, \tau)]^{2}}{2[{\Phi}(t) - {\Phi}(\tau)]}\right\} \bar{{\Psi}}_{x}^{\prime}(a_{i}, t).\\ \end{array} $$

In Eqs. A7 and A8, the notation $\bar {{\Psi }}_{x}^{\prime }(a_{i}, t)$ should be interpreted to mean $\bar {{\Psi }}_{x}^{\prime }(x, t)|_{x = a_{i}}$. Together, Eqs. A7 and A8 give the kernel function of the integral equations that allow the first-passage time densities to be computed.

For the time-varying model of Eq. 4, the drift rate is *μ*(*x*,*t*) = *μ**𝜃*(*t*), which depends on time but not state, and the diffusion coefficient is $\sigma ^{2}(t) = {\sigma _{1}^{2}}\theta (t) + {\sigma _{2}^{2}}$. For this process, Eq. A4 takes the form

A9 $$ \mu\theta(t) = \frac{\sqrt{{\sigma_{1}^{2}}\theta(t) + {\sigma_{2}^{2}}}}{2}c_{1}(t) + \frac{x}{2}\left[c_{2}(t) + \frac{{\sigma_{1}^{2}}\theta^{\prime}(t)}{{\sigma_{1}^{2}}\theta(t) + {\sigma_{2}^{2}}}\right].  $$

Because the left-hand side of Eq. A9 does not depend on *x*, the term in square brackets on the right-hand side must be zero to make it an identity. We therefore obtain

$$ c_{1}(t) = \frac{2\mu\theta(t)}{\sqrt{{\sigma_{1}^{2}}\theta(t) + \sigma^{2}}} $$

and

$$ c_{2}(t) = -\frac{{\sigma_{1}^{2}}\theta^{\prime}(t)}{{\sigma_{1}^{2}}\theta(t) + \sigma^{2}}. $$

To evaluate Eqs. A5 and A6, we need the integral of *c*_2_(*t*), which is -${\log [\sigma _{1}^{2}}\theta (t) + {\sigma _{1}^{2}}]$. (We omit the constant of integration because the expression for the kernel involves differences of functions evaluated at two different points in time from which constants of integration drop out.) Substituting this expression into Eqs. A5 and A6 and simplifying yields

A10 $$ \bar{{\Psi}}(x,t) = x - \mu {\int}^{t} \theta(s)  ds  $$

and

A11 $$ {\Phi}(t) = {\sigma_{1}^{2}}{\int}^{t} \theta(s) ds + {\sigma_{2}^{2}} t.  $$

Substituting these expressions into Eq. A8 and evaluating $\bar {{\Psi }}(x,t)$ at *x* = *a*_*i*_ and *x* = *a*_*j*_ yields the kernel of the integral equation

A12 $$ \begin{array}{@{}rcl@{}} {\Psi}(a_{i},  t|  a_{j},  \tau) & = & \frac{1}{\sqrt{2\pi[ {\sigma_{1}^{2}}{\int}_{\tau}^{t} \theta(s) ds + {\sigma_{2}^{2}}(t - \tau)]}}\\ &&\exp\left\{-\frac{\left[a_{i} - a_{j} - \mu {\int}_{\tau}^{t} \theta(s) ds\right]^{2}} {2[{\sigma_{1}^{2}}{\int}_{\tau}^{t} \theta(s) ds + {\sigma_{2}^{2}}(t - \tau)]}\right\} \\ &&\times \frac{1}{2}\left\{-\mu\theta(t) - \frac{a_{i} - a_{j} - \mu {\int}_{\tau}^{t} \theta(s)  ds} {[{\sigma_{1}^{2}}{\int}_{\tau}^{t} \theta(s) ds + {\sigma_{2}^{2}}(t - \tau) ]}[{\sigma_{1}^{2}}\theta(t) + {\sigma_{2}^{2}}]\right\},\!\\ \end{array} $$

which is Eq. 7 in the text. Equation A12 used in Eqs. 5 and 6 gives the first-passage time probability densities *g*_*A*_(*a*_1_,*t*|*z*, 0) and *g*_*B*_(*a*_1_,*t*|*z*, 0), which are the predicted joint decision-time densities in the model.

To evaluate Eqs. 5 and 6 numerically, we discretize them and evaluate them on the mesh *k*Δ, *k* = 1, 2,…. The discretized forms of the equations (Buonocore et al.,, 1990, Smith, 2000, Equations 47a and 47b) are

A13 $$ \begin{array}{@{}rcl@{}} g_{A}(a_{1}, k{\Delta} | z, 0) & = & -2{\Psi}(a_{1}, k{\Delta} | z, 0) \\ && + 2{\Delta}\sum\limits_{j=1}^{k-1} g_{A}(a_{1}, j{\Delta} | z, 0) {\Psi}(a_{1}, k{\Delta} | a_{1}, j{\Delta}) \\ && + 2{\Delta}\sum\limits_{j=1}^{k-1} g_{B}(a_{2}, j{\Delta} | z, 0) {\Psi}(a_{1}, k{\Delta} | a_{2}, j{\Delta}),\!\\ \end{array} $$

and

A14 $$ \begin{array}{@{}rcl@{}} g_{B}(a_{2}, k{\Delta} | z, 0) & = & 2{\Psi}(a_{2}, k{\Delta} | z, 0) \\ && - 2{\Delta}\sum\limits_{j=1}^{k-1} g_{A}(a_{1}, j{\Delta} | z, 0) {\Psi}(a_{2}, k{\Delta} | a_{1}, j{\Delta}) \\ && - 2{\Delta}\sum\limits_{j=1}^{k-1} g_{B}(a_{2}, j{\Delta} | z, 0) {\Psi}(a_{2}, k{\Delta} | a_{2}, j{\Delta}),\!\\ \end{array} $$

for *k* = 2, 3,…. For *k* = 1, the equations reduce to

A15 $$ g_{A}(a_{1}, {\Delta} | z, 0) = -2{\Psi}(a_{1}, {\Delta} | z, 0)  $$

and

A16 $$ g_{B}(a_{2}, {\Delta} | z, 0) = 2{\Psi}(a_{2}, {\Delta} | z, 0).  $$

Equations A13 and A14 represent the first-passage time densities at time *k*Δ as functions of their values at preceding times *j*Δ, *j* < *k*, and of the kernel function Eq. A8. Buonocore et al., (1987) proved that if the kernel is chosen according to Eq. A8, then the discrete approximations converge to the true first-passage densities as ${\Delta } \rightarrow 0$. Equations A13 to A16 provide a computationally efficient and numerically stable way to obtain predictions for a model with time-varying drift and diffusion rates. Voskuilen et al. (2016, Appendix B) gave complementary expressions for obtaining first-passage time densities for a Wiener diffusion process with constant drift and diffusion rates through time-varying boundaries, which they used to evaluate collapsing-bounds models.

## Appendix B: Parameterization of the models

In this appendix we describe the way in which we parameterized the standard and time-varying diffusion models in fitting them to data. The effects of bias in the collapsed Dutilh et al., (2019) data set were represented by a three-level factor that describes whether stimuli were presented with low, equal, or high frequency. Response bias in the standard diffusion model is represented by the starting point for evidence accumulation, *z*. An unbiased decision-maker will set the starting point equidistantly between the decision boundaries, *z* = *a*/2, whereas a decision-maker who is biased towards one of the responses will set the starting point closer to the associated boundary so that the response is made with less evidence. Collapsing the data over left and right responses as was done in the Dutilh et al., (2019) study implies a symmetry constraint on response bias: If the bias towards the correct response for high-frequency stimuli is *z*, then the bias towards the correct response for low-frequency stimuli must be − *z*. To further reduce the number of bias parameters, we expressed starting point as a proportion of the distance between the two boundaries, *a*. To do so, we defined a relative starting point parameter, *π*_*z*_, by the relation *z* = (1 + *π*_*z*_)*a*/2. The parameter *π*_*z*_ varies between − 1 and + 1, with *π*_*z*_ = 0 corresponding to *z* = *a*/2, which represents the absence of bias. The advantage of parameterizing bias in relative rather than absolute terms is that a single parameter can be used to represent response bias with different boundary separations.

In addition to response bias, many studies have found evidence for stimulus bias, in which evidence for the two responses accumulates at unequal rates, as first proposed by Ashby (1983). Stimulus biases are often found in tasks like recognition memory, in which evidence for old and new items accumulate at different rates (Ratcliff and Smith, 2004; Starns et al., 2012). In its most general form, stimulus bias requires two drift rate parameters per discriminability condition to represent it, but it can often be modeled with fewer parameters by using a *drift criterion*, *c*_*ν*_, which assumes that unequal drift rates are the result of a stimulus bias process whose effects are constant across discriminability levels. Specifically, if *ν*_*A*_ and *ν*_*B*_ are the drift rates for the two stimuli, then the drift criterion model assumes that *ν*_*A*_ = *ν* − *c*_*ν*_ and *ν*_*B*_ = −*ν* + *c*_*ν*_. When there is only one discriminability level in the experiment this representation does not result in any net savings in the number of free parameters, but when there are *k* conditions in the experiment, it allows drift rate to be parameterized with *k* + 1 rather than 2*k* parameters. Along with response bias, we investigated whether varying the relative frequencies of two stimuli within a block affected the rates at which evidence accumulated for the two responses. To do so, we assumed that there were two mean drift rates parameters, *ν*(*h*) and *ν*(*e*), for hard and easy stimuli. With the addition of a drift criterion, the drift rates for hard and easy, high, equal, and low frequency stimuli were *ν*(*h*) − *c*_*ν*_, *ν*(*e*) − *c*_*ν*_, *ν*(*h*), *ν*(*e*), *ν*(*h*) + *c*_*ν*_, and *ν*(*e*) + *c*_*ν*_, respectively. Of the teams who fit the full diffusion model in the Dutilh et al., (2019) study, only one of them (Trueblood, Holmes, & Visser) appears to have considered models with drift rate bias.

To complete the models, we assumed that there were two boundary separation parameters, *a*(*s*) and *a*(*a*), for speed and accuracy conditions, a single drift rate variability parameter, *η*, two starting point variability parameters for speed and accuracy conditions, *s*_*z*_(*s*) and *s*_*z*_(*a*), a mean nondecision time, *T*_er_, and a nondecision time variability parameter, *s*_*t*_. We made the usual assumptions that drift rates were normally distributed and starting points and nondecision times were uniformly distributed. For these latter sources of variability, *s*_*z*_ and *s*_*t*_ denote the ranges of the uniform distributions. To facilitate investigation of selective influence, we parameterized the distribution of nondecision times by its leading edge rather than by its midpoint. Under this parameterization the average nondecision time for models in which nondecision time variability was nonzero was *T*_er_ + *s*_*t*_/2. For the time-varying models, there were three additional parameters: the rate and shape parameters, *β* and *n*, of the growth rate function *𝜃*(*t*) in Eq. 9 and the premature sampling noise parameter, *σ*^2^, in Eq. 4.

To fit the models to data we assumed that RT was the sum of independent decision time and nondecision time random variables and that drift rate, *μ*, was normally distributed across trials with mean *ν* and standard deviation *η*. We obtained predictions for models with drift rate and nondecision time variability by numerically integrating Eqs. A13 and A14 across the distribution of *μ* and then numerically convolving the marginal joint distributions with the discretized distribution of nondecision times. Because mixing (numerical integration) and convolution are commutative, the order in which they are performed is immaterial, so we only needed to compute the convolution once for each marginal distribution rather than for each value of the integrand.

## Footnotes

1 Because *T*_er_ comprises both predecisional and postdecisional times, the range 300 to 600 ms in Fig. 1A of Matzke and Wagenmakers (2009) should be viewed as an upper bound on the onset of evidence accumulation rather than the onset time itself.

2 Readers who are unfamiliar with stochastic differential equations, including the reason for writing them in differential form rather in the more familiar form involving derivatives and the reason why their behavior does not follow the normal rules of calculus, may find a tutorial introduction aimed at the study of decision processes in Smith (2000).

3 The sum of two independent standard Brownian motions, each with unit variance, is a Brownian motion with variance 2. If $\sigma _{1}^{\prime }\sqrt {\theta (t)}$ and $\sigma _{2}^{\prime }$ are the infinitesimal standard deviations of the individual processes in Eq. 3, then the coefficients of the coactive process in Eq. 4 will be $\sigma _{1}\sqrt {\theta (t)} = \sigma _{1}^{\prime }\sqrt {\theta (t)}/\sqrt {2}$ and $\sigma _{2} =\sigma _{2}^{\prime }/\sqrt {2}$. In the text we use the same notation for the coefficients of both forms of the process, without using primes to distinguish them.

4 The 280 ms fast-guess exclusion criterion was suggested to us by Roger Ratcliff (personal communication, October 31, 2019). Although it can be argued that responses in the range 200-280 ms are valid and should be retained, we chose to use the higher cutoff to forestall the criticism that the comparatively poor performance of the standard diffusion model in our study was an artifact of inappropriate data screening.

5 We used the property that *𝜃*(*t*) approaches a step function when *β*/*n* becomes large to validate the code for the time-varying model. With model parameters chosen in this way, the *G*^2^ values for the standard and time-varying models become progressively more similar to each other and indistinguishable in the limit.
